# Comparison of LC-MS-based methods for the determination of carboxylic acids in animal matrices

**DOI:** 10.1007/s00216-023-05113-8

**Published:** 2024-01-05

**Authors:** Heidi E. Schwartz-Zimmermann, Manuel Hündler, Nicole Reiterer, Sara Ricci, Raul Rivera-Chacon, Ezequias Castillo-Lopez, Qendrim Zebeli, Franz Berthiller

**Affiliations:** 1 Department of Agrobiotechnology, IFA-Tulln, Institute of Bioanalytics and Agro-Metabolomics, University of Natural Resources and Life Sciences, Vienna (BOKU), Tulln, Austria; 2 Christian Doppler Laboratory for Innovative Gut Health Concepts of Livestock, Vienna, Austria; 3 Pfizer Austria, Orth an der Donau, Austria; 4 Marinomed Biotech AG, Korneuburg, Austria; 5 Department for Farm Animals and Veterinary Public Health, Institute of Animal Nutrition and Functional Plant Compounds, University of Veterinary Medicine Vienna, Vienna, Austria

**Keywords:** Derivatization, Reversed-phase high-performance liquid chromatography, Anion exchange chromatography, Mass spectrometry, Short-chain fatty acids

## Abstract

Carboxylic acids (CAs) are key players in human and animal metabolism. As they are hardly retained under reversed-phase liquid chromatography (RP-LC) conditions in their native form, derivatization is an option to make them accessible to RP-LC and simultaneously increase their response for mass spectrometric detection. In this work, two RP-LC tandem mass spectrometry-based methods using aniline or 3-nitrophenylhydrazine (3-NPH) as derivatization agents were compared with respect to several factors including completeness of derivatization, apparent recoveries (*R*_*A*_s) in both cow feces and ruminal fluid, and concentrations obtained in feces and ruminal fluid of cows. Anion exchange chromatography coupled to high-resolution mass spectrometry (AIC-HR-MS) served as reference method. Derivatization efficiencies were close to 100% for 3-NPH derivatization but variable (20–100%) and different in solvent solutions and matrix extracts for aniline derivatization. Likewise, average *R*_*A*_s of ^13^C-labeled short-chain fatty acids as internal standards were around 100% for 3-NPH derivatization but only 45% for aniline derivatization. Quantification of CAs in feces and ruminal fluid of cows initially fed a forage-only diet and then transitioned to a 65% high-grain diet which yielded similar concentrations for 3-NPH derivatization and AIC-HR-MS, but concentrations determined by aniline derivatization were on average five times lower. For these reasons, derivatization with aniline is not recommended for the quantitative analysis of CAs in animal samples.

**Graphical abstract:**

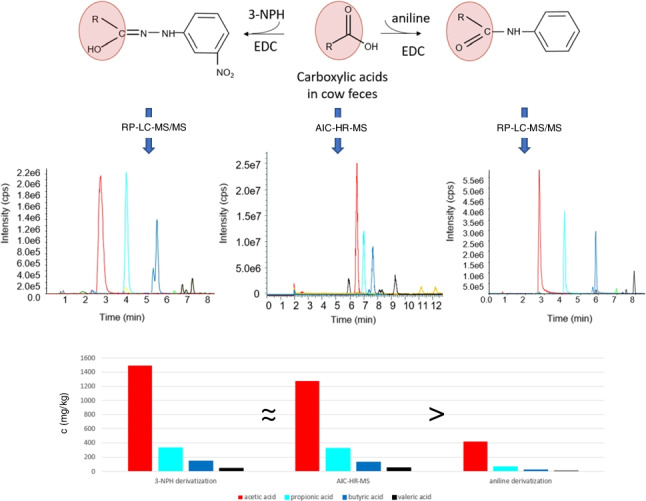

**Supplementary Information:**

The online version contains supplementary material available at 10.1007/s00216-023-05113-8.

## Introduction

Carboxylic acids (CAs) are organic compounds with at least one carboxylic group. The compound class of CAs is diverse and encompasses saturated and unsaturated straight- and branched-chain fatty acids of different chain lengths, keto acids, hydroxy acids, and aromatic CAs as well as di- and tricarboxylic acids. CAs are crucial for energy and mitochondrial metabolism, acid–base regulation, carbohydrate, and lipid and amino acid synthesis as well as in immunity and signaling as summarized by Yang and co-workers [1].

In particular, short-chain fatty acids (SCFAs) are key analytes and mainly produced by anaerobic microbial fermentation of complex carbohydrates such as fiber and starches that occurs mainly in the rumen in ruminants or hindgut in monogastric farm animals. The interplay between the gut microbiome, the animal host, and the diet has become an increasingly hot research topic. In addition, also degradation of starch by ruminal bacteria results in formation of SCFAs, via glucose and pyruvate as intermediates [2]. An excess of dietary starch often leads to an excess of SCFAs, which might result in a pathological state called subacute ruminal acidosis [3]. This typically happens when dairy cows are fed grain-rich diets to meet the increased energy demands. Several studies investigated the metabolic changes upon high-concentrate feeding and reported, in addition to increased levels of SCFAs, also changes in various other metabolites like biogenic amines, amino acids, nucleobases, and other CAs [4–6].

The methods used for the determination of CAs in ruminant matrices are ^1^H-NMR spectroscopy [4] and gas chromatography coupled to mass spectrometry (GC-MS) [6] as well as anion exchange chromatography coupled to mass spectrometry (AIC-MS) [5]. ^1^H-NMR spectroscopy, while being a universal and widely used technique in metabolomics studies, offers lower sensitivity than mass spectrometric methods. AIC-MS is a versatile technique for the quantitative analysis of CAs, e.g., [5, 7], but less widespread than other chromatographic mass spectrometric techniques like reversed-phase high-performance liquid chromatography coupled to mass spectrometry (RP-LC-MS) and GC-MS. Whereas classical RP stationary phases hardly retain native SCFAs with ≤ 5 carbon atoms [8], selected SCFAs containing between 4 and 6 carbon atoms could be detected by hydrophilic interaction chromatography (HILIC) coupled with tandem [8] or high-resolution mass spectrometry [9]. As native SCFAs ionize poorly and lack specific selected reaction monitoring (SRM) transitions due to poor fragmentation, bad detectability could be the reason why only selected SCFAs were detectable by HILIC coupled with MS [9].

Recently, several RP-LC-MS/MS-based methods for the determination of CAs have been published, e.g., [10–18]. These methods employ derivatization of the carboxylic group, which on the one hand reduces the analyte’s polarity, making it accessible to RP-HPLC separation, and on the other hand improves the ionization of the compounds. Several different derivatization reagents were used, including aniline [10, 18], 3-nitrophenylhydrazine (3-NPH) [11, 17], and dansylhydrazine (DnsHz) [12, 16], which are all used in combination with 1-ethyl-3-(3-dimethylaminopropyl)carbodiimide (EDC). These three reagents are commercially available also in ^13^C-labeled form, allowing the in-house production of internal standards and the application of chemical isotope labeling techniques, e.g., [12, 16]. p-Dimethylaminophenacyl [13], *N*,*N*-dimethyl-6,7-dihydro-5H-pyrrolo[3,4-d]pyrimidin-2-amine [14], and 2-dimethylaminoethylamine [15] that were also used as derivatization agents are not commercially available in labeled form, so that chemical synthesis is required. Despite the benefits of derivatization, several uncertainty factors are associated with derivatization methods. They range from completeness of the derivatization reaction, especially in the presence of a matrix, to the selectivity of the chosen labeling reagent and the stability of the derivatized compounds.

**Fig. 1:**
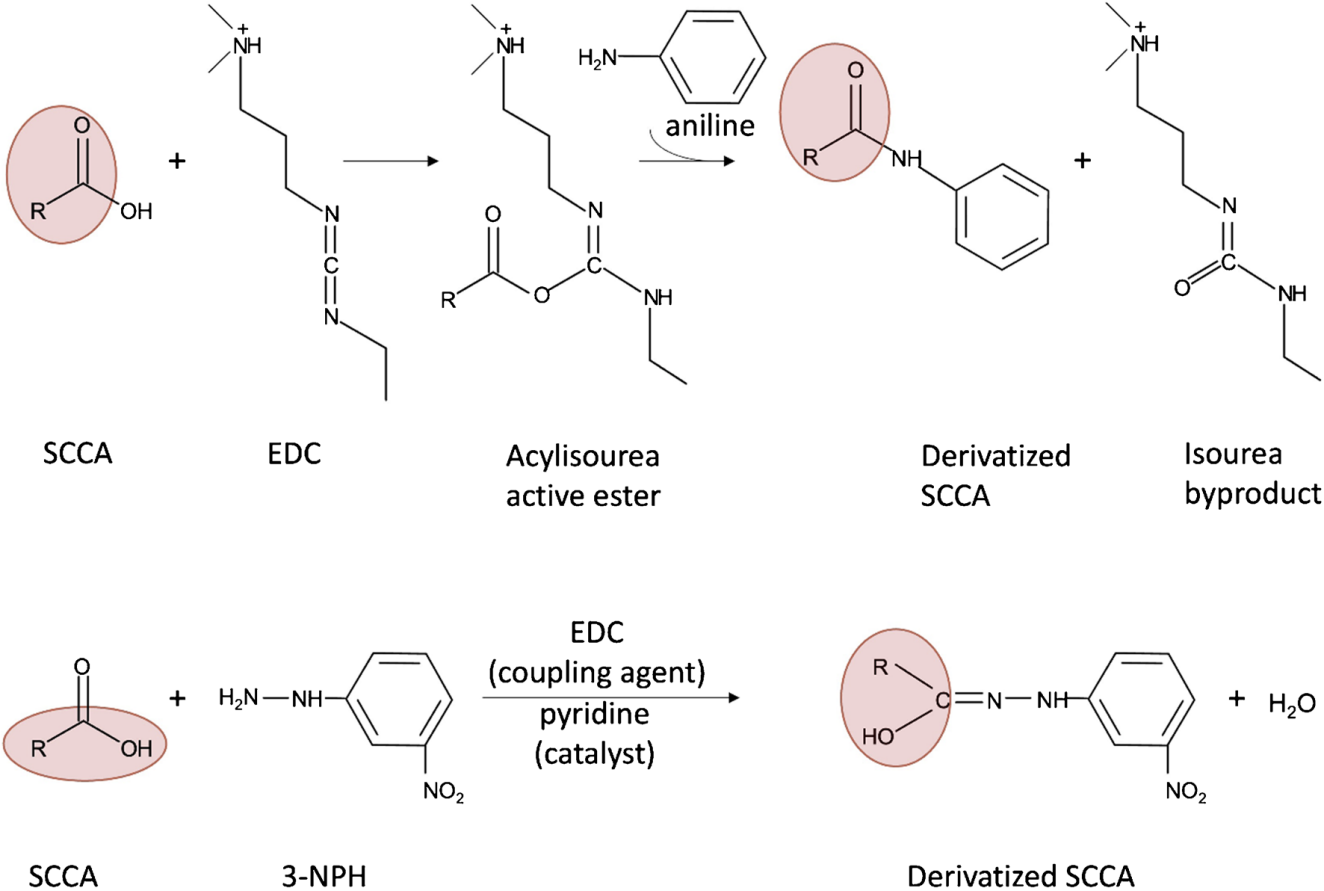
Derivatization of short-chain carboxylic acids (SCCAs) with aniline and 3-nitrophenylhydrazine (3-NPH) using 1-ethyl-3-(3-dimethylaminopropyl)carbodiimide (EDC) as cross-linker

In the present work, we aim to compare two specific methods for derivatization of CAs that are based on previously published protocols [10, 11] in order to find the best suited method for quantification of CAs in animal samples. Analyte coverage, completeness of derivatization reaction, apparent recoveries in different animal matrices, limits of detection and quantification, concentrations obtained in feces and ruminal fluid of cows, and ease of performance will be evaluated. Chan and co-workers [10] performed a sequential derivatization of CAs with EDC and aniline, whereas Han and co-workers [11] used EDC and 3-nitrophenylhydrazine (3-NPH) as labeling reagent (Fig. 1). Not relying on derivatization, we decided on AIC-HR-MS as reference method for comparison. As the two derivatization methods were developed for analysis of CAs in human feces, we partly optimized the protocols for application to animal matrices with higher analyte concentrations. The comparison of three different methods will assess their reliability and evaluate their suitability for metabolome profiling. Most importantly, we verified that not all previously published methods are equally suitable to analyze samples of biological origin for CAs.

## Materials and methods

### Reagents, standards, and standard solutions

Acetonitrile (ACN, LC-MS grade) was purchased from VWR International GmbH (Vienna, Austria), methanol (LC-MS grade) and formic acid (MS grade) were obtained from Honeywell (Vienna, Austria), and aqueous ammonium solution (25%, p.a.) was from Merck (Darmstadt, Germany). Aniline was purchased from Acros Organics (Vienna, Austria),^13^C_6_-aniline from Toronto Research Chemicals (Toronto, Canada), 3-nitrophenylhydrazine (3-NPH), pyridine, and β-mercaptoethanol from Sigma-Aldrich (Vienna, Austria), while ^13^C_6_-3-nitrophenylhydrazine was supplied by Cayman Chemical (Vienna, Austria). 1-(3-Dimethylaminopropyl)-3-ethyl-carbodiimide hydrochloride (EDC) was obtained from VWR. Water purified with an ultrapure water system (Sartorius arium pro, Göttingen, Germany) was used in all experiments.

Carboxylic acid reference standards were purchased from Sigma-Aldrich, VWR, Merck, TCI-Germany (Eschborn, Germany), Acros Organics, and Extrasynthese (Genay, France). Information on the standards acquired and their respective manufacturers is given in the electronic supplementary material. Stock solutions of fatty acids with ≥ 10 carbon atoms were prepared in ACN, whereas all other reference standards were dissolved in ACN/water (50/50, *v*/*v*). The concentration of single stock solutions of CAs was 1000 mg/L, and 10 mg/L dilutions of the single stocks were prepared in ACN/water (50/50, *v*/*v*) for testing the derivatization efficiency. The preparation of mixed stock solutions for establishing calibration curves and for validation experiments is described in the electronic supplementary material (Table S1).

### Animal samples

The feces and ruminal fluid samples used for method validation were obtained from a healthy rumen-cannulated cow. Samples for investigating the changes in CA concentrations in feces and ruminal fluid of cows fed high-concentrate diet were obtained from a larger animal experiment performed at the dairy research farm of University of Veterinary Medicine, Vienna. This animal trial is described in detail in [5, 19]. In the sub-experiment described here, five Holstein non-lactating rumen-cannulated cows were initially fed a forage-only diet. After the first week of the trial, the concentrate proportion (including a.o. 30% barley, 18% triticale, 23% bakery by-product, and 24% rapeseed meal) in the feed was gradually increased to 65% over the course of 7 days and kept at 65% for 4 weeks. Feed and water were available *ad libitum*. Samples of feces and ruminal fluid were collected once a week during the trial 4 h after feeding and stored at −80 °C until analysis. Prior to analysis, 200 mg aliquots of all five feces and ruminal fluid samples of week 1 (forage feeding), week 3 (first week of 65% concentrate feeding), and week 5 (third week of concentrate feeding) were combined to prepare three pooled samples of each matrix.

### Derivatization of carboxylic acids

#### Derivatization conditions

Derivatization with 3-NPH as published by Han et al. [11] started by mixing 40 μL of standard solution or sample extract (diluted 1 + 1 with ACN/water (*v* + *v*), see below for preparation) with 20 μL of 200 mM 3-NPH and 20 μL of 120 mM EDC solution containing 6% pyridine (all prepared in ACN/water (50/50, *v*/*v*)). This mixture was held at 40 °C for 30 min, subsequently diluted to 1.4 mL with ACN/water (50/50, *v*/*v*), and finally centrifuged at 14,350 rcf for 10 min prior to LC-MS/MS measurement.

The derivatization protocol with aniline is based on the published method of Chan et al. [10], albeit with some modifications, most importantly to adjust the derivatization reagent concentration to animal sample matrices. In our work, a 90 μL aliquot of standard solution or sample extract was mixed with 5 μL of 1000 mM aniline and 50 μL of 100 mM EDC (both prepared in ACN/water, 50/50, *v*/*v*) to reach 35 mM of aniline and EDC in the derivatization solution. This mixture was incubated at 4 °C for 2 h and subsequently quenched with 50 μL of 100 mM formic acid (equimolar to the original concentration of aniline and EDC in derivatization solution) and 50 μL of 18.5 mM 2-mercaptoethanol at 4 °C for 2 h. Finally, the solution was diluted to 1225 µL with ACN/water (50/50, *v*/*v*) and centrifuged for 10 min prior to LC-MS/MS measurement.

#### Derivatization efficiency

To evaluate the derivatization efficiency of both protocols for a variety of CAs, 10 mg/L solutions of all CAs listed in “reagents, standards, and standard solutions” were prepared in ACN/water (50/50, *v*/*v*) and derivatized with 3-NPH and aniline, respectively, as described above. After derivatization, the samples were measured by RP-HPLC-MS/MS (see below). In addition, the derivatized samples were measured by AIC-HR-MS (see below) to evaluate the completeness of the derivatization reaction. The non-derivatized portion was quantified on the basis of external calibration curves established for all investigated CAs in a concentration range between 3 and 1000 ng/mL. Additionally, a fecal and a ruminal fluid extract prepared as described in “Extraction of feces and ruminal fluid” were derivatized as described above and measured by AIC-HR-MS to assess the influence of the matrix on the derivatization efficiency.

#### Testing parameters for derivatization of carboxylic acids with aniline

To investigate the ruggedness of the derivatization method with aniline, a mixed standard solution containing 6 mg/L of acetic acid and 3 mg/L of propionic acid, butyric acid, valeric acid, hexanoic acid, iso-butyric acid, iso-valeric acid, and 3-phenylpropionic acid as well as a ruminal fluid extract were derivatized in triplicate as described above with the following modifications: (a) no modification compared to the derivatization conditions described above, (b) derivatization at 40 °C for 2 h, and (c) derivatization as described above but threefold increase in the concentration of aniline and ECD. In addition, ruminal fluid extracts prepared by shaking 20 µL of ruminal fluid with 80 and 2480 µL, respectively, of ACN/water (80/20, *v*/*v*) were derivatized as described above. The derivatized concentrated extracts were measured directly and after 1 + 4 (*v* + *v*) dilution. The diluted extracts were originally brought to 245 µL and measured as such as well as after 1 + 4 (*v* + *v*) dilution to 1225 µL. For determination of the highly concentrated fatty acids, all derivatized extracts were also measured after 1 + 9 (*v* + *v*) dilution. CAs were quantified on the basis of calibration curves derivatized as described in “derivatization conditions” and established between 0.001 and 1.22 mg/L in measurement solution. Differences between derivatization as described above in “derivatization conditions” and derivatization at 40 °C or threefold reagent concentration were determined in paired sample *t*-tests at α = 0.05.

#### Production of ^13^C-labeled injection standards

^13^C_6_-Anilin and ^13^C_6_-3-NPH injection standard mixes were produced for the metabolites that could be detected in feces and/or ruminal fluid. Their composition and concentrations are given in the Online Resource Table S2. The derivatization conditions were the same as described above with the following exceptions: ^13^C_6_-anilin and ^13^C_6_-3-NPH were used as derivatization reagents, only 45 µL aliquots of the stocks were used for derivatization with ^13^C_6_-anilin, and the final volume of the derivatization approach with ^13^C_6_-3-NPH was 1090 µL to achieve the same concentration as obtained with the ^13^C_6_-anilin derivatization. The derivatization was checked by 1:30 (v:v) dilution of the produced injection standard mix and subsequent HPLC-MS/MS measurement. Long-term storage of injection standard mixes was at −80 °C, whereas aliquots of the mixes for daily use were stored at −20 °C.

### Liquid chromatographic mass spectrometric determination of carboxylic acids

#### UHPLC-MS/MS determination of derivatized carboxylic acids

UHPLC-MS/MS analyses were carried out on an Agilent 1290 series UHPLC system (Agilent Technologies, Waldbronn, Germany) coupled to a SCIEX Triple Quad 5500+ mass spectrometer equipped with a Turbo V ion source (SCIEX, Foster City, CA, USA). Derivatized compounds were separated on an Agilent SB-C18 RRHD column (100 × 2.1 mm, 1.8 μm particle size) at a flow rate of 0.4 mL/min and at 40 °C. The injection volume was 1 μL. Mobile phases A and B were water and acetonitrile, respectively, both containing 0.1% formic acid (*v*/*v*). Compounds derivatized with 3-NPH were separated with the following linear gradient: 0.0–0.5 min, 15% B; 0.5–10 min, linear gradient to 50% B; 10–11 min, linear gradient to 100% B; 11–13 min, 100% B; and 13.1–16 min, 15% B. The linear gradient for separation of compounds derivatized with aniline was the same as the gradient for compounds derivatized with 3-NPH except that 100% B was held until 14 min, prolonging the runtime by 1 min to 17 min. The electrospray (ESI) source was operated at 450 °C and −4500 V (negative mode for 3-NPH derivatives) or 4500 V (positive mode for aniline derivatives). Ion source gas 1 and 2 were set to 70 psi. SRM parameters used for derivatization checks and for the final methods, respectively, as well as retention times of derivatized compounds are given in the Online Resource Tables S3 and S4. Analyst® version 1.7.1 (Sciex) was employed for instrument control and data evaluation.

#### Anion exchange chromatography high-resolution mass spectrometry

A Dionex Integrion HPIC system (Thermo Fisher Scientific, Vienna, Austria) was used for anion exchange chromatography (AIC). Separation was carried out on a Dionex IonPac AS11-HC column (250 × 2 mm, 4 μm particle size, Thermo Scientific) protected by a Dionex IonPac AG11-HC guard column (50 × 2 mm, 4 μm) at 30 °C and at a flow rate of 0.38 mL/min. The injection volume was 2 µL. The following potassium hydroxide gradient was produced by an eluent generator equipped with a KOH cartridge: 1 mM KOH for 2 min, 1–10 mM from 2 to 10 min, 10–25 mM from 10 to 15 min, 25–80 mM from 15 to 20 min, isocratic period at 80 mM from 20 to 26 min, and re-equilibration at 1 mM from 26 to 28 min. After separation, a Dionex AERS 500, 2-mm suppressor operated at 76 mA, was used to replace potassium by protons. To facilitate ionization, methanol was added to the mobile phase at a flow rate of 0.06 mL/min after passing the suppressor.

Mass spectrometric detection was carried out on a Thermo Scientific Q Exactive Orbitrap mass spectrometer operated in full scan mode (*m/z* 50–750) at a resolution of 70,000 using ESI in negative mode. The ESI source parameters were spray voltage 3 kV, capillary temperature 230 °C, sheath gas 50, auxiliary gas 13, sweep gas 3, S-Lens RF level 45, and auxiliary gas heater 430 °C. The automatic gain control target was set to 1 × 10^6^ ions, and 200 ms was used as maximum injection time. The mass-to-charge ratios of the deprotonated compounds and the retention times are given in the Online Resource Table S5. Thermo Xcalibur^TM^ software was employed for instrument control (version 4.1.31.9) and data evaluation (version 4.4.16.14).

### Extraction of feces and ruminal fluid

Before extraction, 100 mg aliquots of wet feces samples were spiked with 100 µL and 20 µL aliquots, of rumen fluid sample were spiked with 50 µL of internal standard (IS) mix containing 500 mg/L ^13^C_2_-acetic acid and 250 mg/L ^13^C_3_-propionic and ^13^C_4_-butyric acid. One hundred mg aliquots of feces were then extracted by shaking with 1.7 mL of ACN/water (80/20, *v*/*v*) at 4 °C for 2 h. Extraction of ruminal fluid was achieved by shaking 20 µL aliquots of ruminal fluid with 430 µL of ACN/water (80/20, *v*/*v*) at 4 °C for 5 min. To investigate the influence of the extraction solvent, quadruplicate extraction of feces and ruminal fluid was also performed with ACN/water (50/50, *v*/*v*), and samples were subsequently derivatized with aniline as described above. All sample extracts were centrifuged at 14,350 rcf and 4 °C for 10 min. To evaluate extraction losses and matrix effects, the IS mix was also spiked into pure extraction solvent at the same concentration as in the sample extracts at 100% recovery.

Prior to derivatization with aniline and 3-NPH, feces and ruminal fluid extracts were diluted 1 + 1 (*v* + *v*) with ACN/water (50/50, *v*/*v*). The feces and ruminal fluid samples derivatized with 3-NPH were measured directly and after 1 + 9 (*v* + *v*) dilution. Feces and ruminal fluid samples derivatized with aniline were measured directly and after 1 + 4 (*v* + *v*) dilution. After derivatization and dilution, but preceding LC-MS/MS measurement, 5 µL aliquots of the respective derivatized injection standard mixes for feces and ruminal fluid were added to 145 µL of derivatized sample solution. Prior to AIC-HR-MS measurement, feces and ruminal fluid extracts were diluted 1 + 19 (*v* + *v*) with ACN/water (50/50, *v*/*v*). Feces sample extracts were additionally measured after 1 + 99 (*v* + *v*) dilution to account for high concentrations of some CAs in the biological samples.

### Method validation and quantification

To determine the limits of detection (LOD) and quantification (LOQ) as well as the linearity of CAs, derivatizable compounds were grouped into three mixes containing 40 mg/L of each compound according to their molecular mass (Online Resource Table S1). After derivatization as described above, albeit with lower dilution factors after derivatization to achieve the highest concentration levels, the concentration range of 3-NPH-derivatized compounds was between 0.0003 and 2.29 mg/L in measurement solution. The calibration curves of the aniline-derivatized compounds ranged between 0.0003 and 11.4 mg/L in measurement solution. For AIC-HR-MS measurement, calibration functions were recorded between 0.001 and 40 mg/L in measurement solution. LODs were determined at a signal-to-noise (S/N) ratio of 3. The minimum S/N ratio for LOQs was 10, and the concentration at the LOQ needed to be within the linear range of the calibration curve.

For the determination of the repeatability, apparent recovery (*R*_*A*_), derivatization yield in matrix compared to pure solvent solutions, and mass spectrometric matrix effects, four 100 mg aliquots of feces were spiked with 100 µL of IS mix (containing 500 mg/L of ^13^C-acetic acid and 250 mg/L each of ^13^C-propionic acid and ^13^C-butyric acid) and subsequently extracted by shaking with 1.7 mL ACN/water (80/20, *v*/*v*) at 4 °C for 1 h. Similarly, four 20 µL aliquots of ruminal fluid were spiked with 50 µL of the same IS mix and extracted with 430 µL of ACN/water (80/20, *v*/*v*) by shaking at 4 °C for 5 min. Aliquots of the supernatants after centrifugation were individually analyzed by each of the three methods for determination of the repeatability and the *R*_*A*_. Each measurement was carried out once. Additionally, 1 mL aliquots of feces extracts and 300 µl aliquots of ruminal fluid extracts were pooled for the determination of derivatization yield and matrix effects. For the determination of *R*_*A*_ of acetic, propionic, and butyric acid in feces/ruminal fluid, 100 / 50 µL of IS mix was also spiked into 1.7 mL/450 µl of pure extraction solvent and worked up and measured in the same way as the samples. *R*_*A*_s were calculated by comparing the peak areas of the labeled standards in derivatized unspiked feces extracts and in derivatized standard solutions.

The derivatization yield in matrix was assessed by standard addition method. To this end, 0.5/1/1.5/2/2.5/3 × of the analyte concentration determined in a pre-experiment was spiked into the pooled extract in duplicate. This was achieved by diluting 250 µl aliquots of the pooled extract with 250 µl of the respective spiking solutions in ACN/water (50/50, *v*/*v*). In parallel, the same volumes of spiking solutions were spiked into 250 µL of ACN/water (80/20, *v*/*v*). The composition of the spiking solutions for feces and ruminal fluid is given in Online Resource Table S6. Aliquots of the spiked extracts and standard solutions were then diluted for measurement by IC-HR-MS (1 + 19, *v* + *v*, for extracts of feces and ruminal fluid and additionally 1 + 99, *v* + *v*) for feces extracts) or derivatized with 3-NPH or aniline as described above. Due to high concentration differences in CAs, feces and ruminal fluid extracts derivatized with 3-NPH were also measured after 1 + 9 (*v* + *v*) dilution, and extracts of feces and ruminal fluid derivatized with aniline were also diluted 1 + 4 (*v* + *v*) prior to measurement.

The derivatization yield in matrix was calculated by dividing the slope of the standard addition curve in matrix extract by the slope of the standard addition curve in pure solvent solution. Matrix effects were assessed by spiking 5 µl of ^13^C-3-NPH or ^13^C-aniline labeled injection standard mix into 145 µl of derivatized extracts, derivatized standard solutions, and pure solvent (ACN/water, 50/50, *v*/*v*). Analysis of the same volume of injection standard mix spiked into 145 µL of pure solvent served as a reference.

### Quantification of CAs in animal samples

Samples for investigating the changes in CA concentrations in feces and ruminal fluid of cows fed high-concentrate diet were worked up and measured once. CAs in extracts of feces and ruminal fluid were quantified on the basis of calibration curves that were established from the spiking mixes given in Online Resource Table S6 and ranged from the LOQ of the individual CAs up to 3 × the analyte concentration in the feces and ruminal fluid samples used for validation. Additionally, standard addition was applied to quantify CAs in feces and ruminal fluid extracts used for method validation. Concentrations in feces and ruminal fluid were determined by applying the respective dilution factors.

## Results and discussion

### Derivatization of carboxylic acids

#### Derivatization conditions

The derivatization procedure used by Chan et al. [10] includes two steps, one derivatization and one quenching step to avoid unfavored reactions within the HPLC system. Stoichiometry calculations indicated the need for higher reagent concentrations than used by Chan et al. due to higher CA concentrations in animal samples compared to human feces samples. A detailed explanation is provided in the Electronic Supplementary Information. In short, Chan et al. used 1 µmol aniline absolute. According to the results presented by Chan and co-workers, the total levels of SCFAs in human stool are in the low nmol/g range. Ruminant feces, however, contains SCFAs in the medium µmol/g range. Based on our extraction protocol, the amount of SCFAs for derivatization is between 0.1 and 1 µmol. Considering previous experiments showing that the reagent concentration has an influence on the derivatization efficiency, we decided to use 5 µmol aniline in the derivatization solution. Since aniline and EDC are consumed stoichiometrically in the derivatization reaction, we used the same molar concentrations of both reagents.

We also modified the quenching step by replacing the potential analyte succinic acid that was used by Chan et al. for quenching, by formic acid which is below the mass cut-off of Orbitrap mass spectrometers. Preliminary experiments with standards in pure solvents yielded no difference in the ability of either formic or succinic acid to quench excess derivatization agents (data not shown). We applied formic acid at the same molar concentration as the derivatization reagents, which allowed us to reduce the concentration of the second quenching agent 2-mercaptoethanol which, according to Chan et al., caused a concentration-dependent depression of peak areas.

The derivatization reaction described by Han et al. [11] used 4 µmol of 3-NPH and 2.4 µmol of EDC per derivatization. As only 20 µL of feces and ruminal fluid extracts were used, the excess of reagent was again at least fivefold. Hence, we applied the derivatization procedure as published by Han and co-workers.

#### Derivatization efficiency

Of the 39 compounds tested, 38 could be derivatized with aniline, albeit with variable derivatization efficiency. Nine compounds (acetic acid, lactic acid, iso-butyric acid, malic acid, succinic acid, 3-hydroxyglutaric acid, malonic acid, fumaric acid, and 10-hydroxydecanoic acid) were fully or at least sufficiently derivatized with a derivatization efficiency over 80%. Thirteen CAs showed a derivatization yield between 50 and 79% (in order of decreasing derivatization yield: 2-methylbutyric acid, butyric acid, 4-hydroxyphenylacetic acid, 3-(3-hydroxyphenyl)propionic acid, 2-ethylbutyric acid, 3-hydroxyphenylacetic acid, phenylacetic acid, iso-valeric acid, 3,3-dimethylbutyric acid, 4-methylvaleric acid, hexanoic acid, 3-methylvaleric acid, and glyceric acid), and six compounds could be derivatized between 20 and 49% (3-phenylpropionic acid, valeric acid, 2-methylvaleric acid, propionic acid, pyroglutamic acid, and 4-hydroxybenzoic acid). Medium- and long-chain fatty acids could also be derivatized, but the derivatization efficiency could not be determined by AIC-HR-MS as fatty acids containing ten or more carbon atoms without polar substituents are not eluted from the used AIC column under the chosen conditions. The benzoic acid derivative, if formed, did not yield a signal upon LC-MS/MS measurement. AIC-HR-MS measurement of the major SCFAs in aniline-derivatized feces and ruminal fluid extracts revealed derivatization efficiencies between 55 and 72% for propionic acid and butyric acid, respectively. Acetic acid was derivatized to 24% in the feces extract and to 60% in the extract of ruminal fluid.

Chan and co-workers [10] had tested the completeness of derivatization of SCFAs (C2-C6, linear and branched, no substituents) by performing a second derivatization step with ^13^C-aniline after the first derivatization and checking for ^13^C-aniline-derivatized SCFAs. As ^13^C-aniline-derivatized SCFAs could not be detected, complete derivatization of all tested SCFAs was assumed. Our results obtained by direct measurement of non-derivatized CAs are in contrast to their findings. The experimental setup by Chan et al. was suitable to investigate whether sufficient derivatization reagent was used. However, other factors affecting the derivatization efficiency were not evaluated, so that the overall derivatization yield could not be assessed in their approach.

Derivatization with 3-NPH was successful with 100% derivatization yield for all compounds measurable by AIC-HR-MS. Medium- and long-chain fatty acids could be derivatized, too. Derivatization efficiencies of acetic, propionic, and butyric acid in feces and ruminal fluid extracts were above 90%. These results are in line with those obtained by Han and co-workers [11], who did not detect free fatty acids after derivatization and obtained basically the same slope of calibration curves prepared by 3-NPH derivatization in matrix and in human fecal sample extract.

As already investigated by Hodek and co-workers [20], dicarboxylic acids were mainly derivatized on both carboxylic acid groups. The most intense precursor ion in MS/MS analysis was the doubly derivatized and singly charged ion. Singly derivatized dicarboxylic acids were also visible in the chromatograms but at much lower intensity. For some dicarboxylic acids, several low-intensity peaks occurred at the same *m/z* ratio of the singly derivatized compound, indicating the formation of derivatives of different structures.

### Liquid chromatographic mass spectrometric determination of carboxylic acids

#### UHPLC-MS/MS determination of derivatized carboxylic acids

**Fig. 2:**
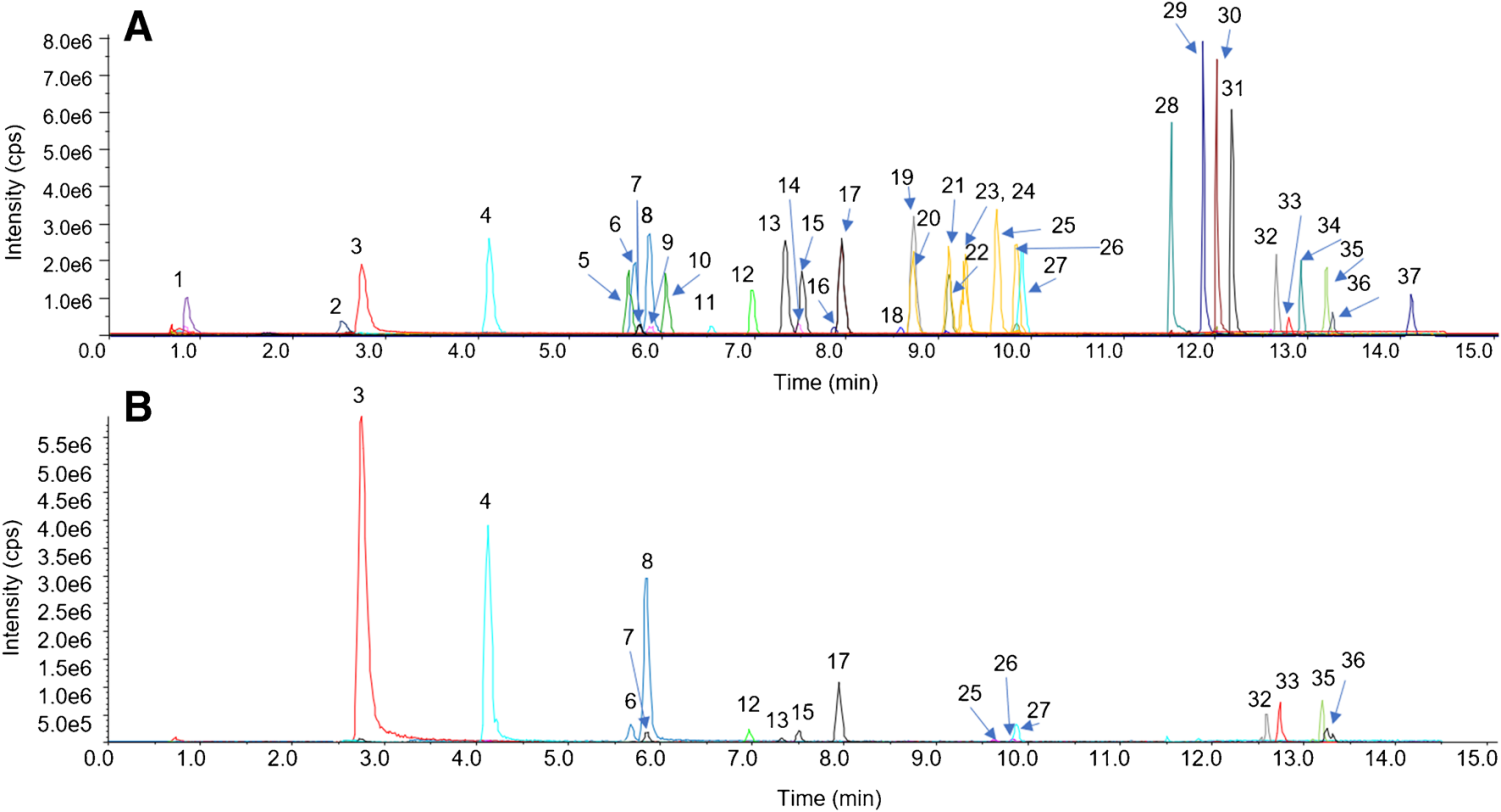
LC-MS/MS chromatogram of carboxylic acids derivatized with aniline in **A** standard solution (300 ng/mL) and **B** feces extract. 1, glyceric acid; 2, lactic acid; 3, acetic acid; 4, propionic acid; 5, 4-hydroxyphenylacetic acid; 6, iso-butyric acid; 7, pyroglutamic acid; 8, butyric acid; 9, 4-hydroxybenzoic acid; 10, 3-hydroxyphenylacetic acid; 11, malic acid (2 × deriv); 12, 3-(3-hydroxyphenyl)propionic acid; 13, 2-methylbutyric acid; 14, succinic acid (2 × deriv); 15, iso-valeric acid; 16, malonic acid (2 × deriv); 17, valeric acid; 18, fumaric acid (2 × deriv); 19, phenylacetic acid; 20, 2-ethylbutyric acid; 21, 3,3-dimethylbutyric acid; 22, 10-hydroxydecanoic acid; 23, 24, 2-methylvaleric acid and 3-methylvaleric acid; 25, 4-methylvaleric acid; 26, hexanoic acid; 27, 3-phenylpropionic acid; 28, caprylic acid; 29, decanoic acid; 30, undecanoic acid; 31, lauric acid; 32, myristic acid; 33, linoleic acid; 34, pentadecanoic acid; 35, palmitic acid; 36, oleic acid; 37, stearic acid

**Fig. 3:**
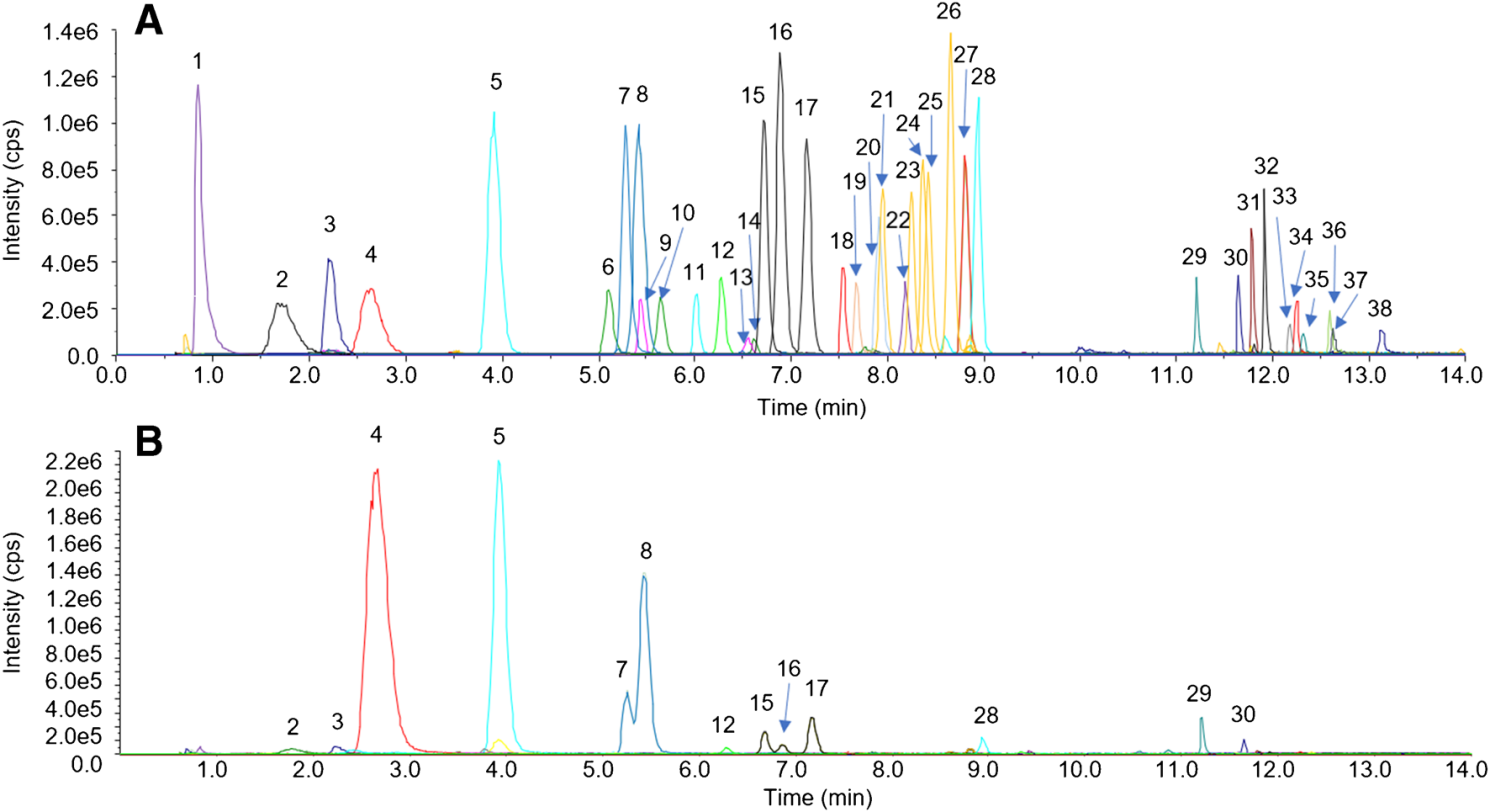
LC-MS/MS chromatogram of carboxylic acids derivatized with 3-nitrophenylhydrazine in **A** standard solution (300 ng/mL) and **B** feces extract. 1, glyceric acid; 2, pyroglutamic acid; 3, lactic acid; 4, acetic acid; 5, propionic acid; 6, 4-hydroxyphenylacetic acid; 7, iso-butyric acid; 8, butyric acid; 9, 4-hydroxybenzoic acid; 10, 3-hydroxyphenylacetic acid; 11, malic acid (2 × deriv); 12, 3-(3-hydroxyphenyl)propionic acid; 13, succinic acid (2 × deriv); 14, malonic acid (2 × deriv); 15, 2-methylbutyric acid; 16, iso-valeric acid; 17, valeric acid; 18, benzoic acid; 19, fumaric acid (2 × deriv); 20, phenylacetic acid; 21, 2-ethylbutyric acid; 22, 10-hydroxydecanoic acid; 23, 3,3-dimethylbutyric acid; 24, 2-methylvaleric acid; 25, 3-methylvaleric acid; 26, 4-methylvaleric acid; 27, hexanoic acid; 28, 3-phenylpropionic acid; 29, caprylic acid; 30, decanoic acid; 31, undecanoic acid; 32, lauric acid; 33, myristic acid; 34, linoleic acid; 35, pentadecanoic acid; 36, palmitic acid; 37, oleic acid; 38, stearic acid

For reasons of comparability, basically the same chromatographic methods were used for separation of CAs derivatized with aniline and 3-NPH, respectively. Chromatograms of derivatized compounds in standard solution and in a fecal extract are shown in Figs. 2 and 3. Derivatization with aniline yielded slightly narrower peaks with better separation of isomeric CAs. In both derivatization approaches, some derivatized CAs were also visible in derivatized blanks. Especially high signals in blanks originated from lactic acid. Hodek and co-workers [20] also detected lactic acid in 3-NPH-derivatized blanks and assumed that it was present already in laboratory equipment or in solvents or reagents used for derivatization. After derivatization with aniline, also medium- and long-chain fatty acids showed carryover with signal intensity tending to increase with increasing chain length.

However, contrary to lactic acid, medium- and long-chain fatty acids showed carryover also into non-derivatized blanks, indicating interactions of derivatized fatty acids with components of the HPLC system.

#### Anion exchange chromatography high-resolution mass spectrometry

**Fig. 4:**
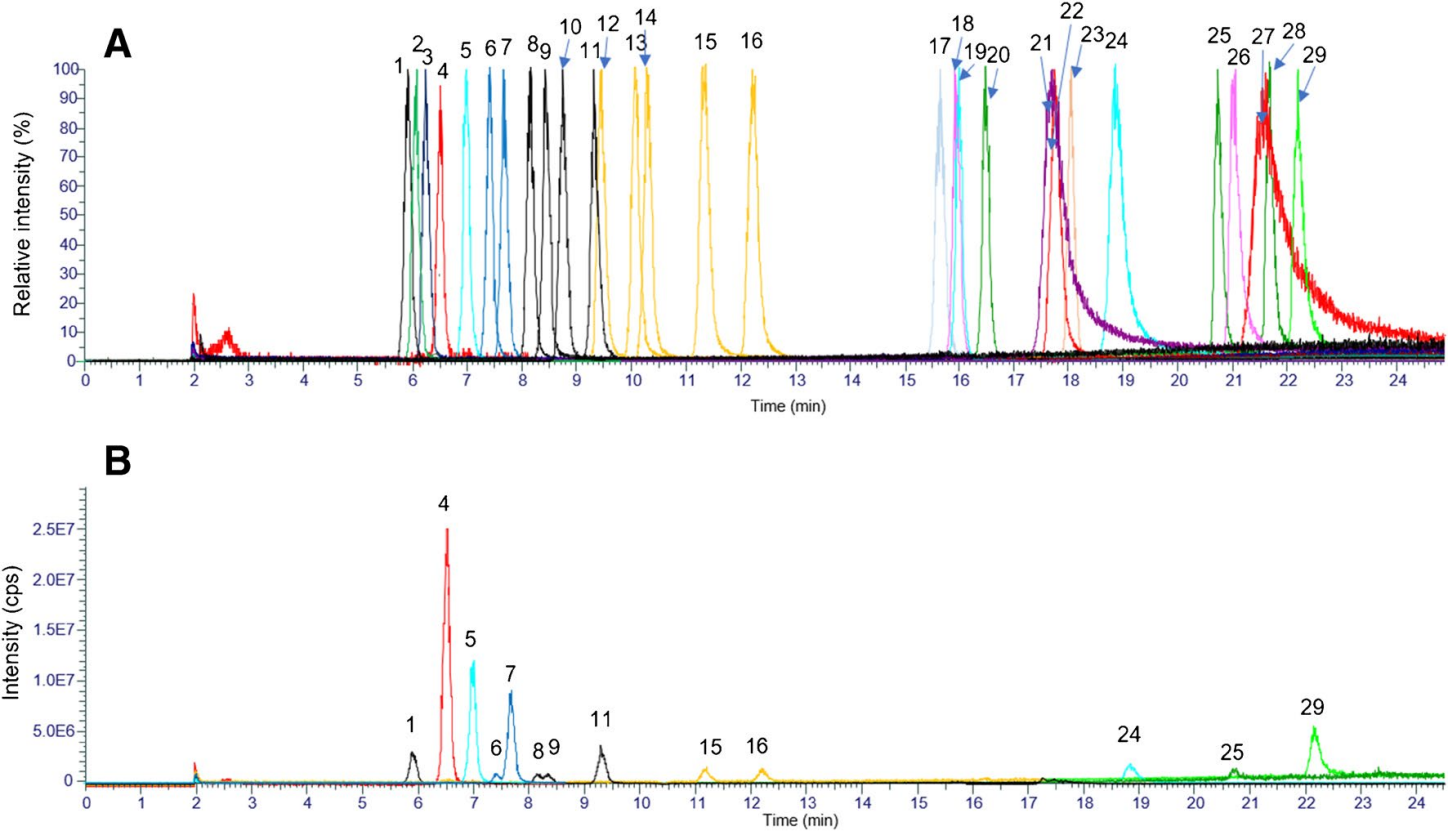
AIC-HR-MS chromatograms of carboxylic acids in **A** standard solution (300 ng/mL, relative intensity) and **B** feces extract (absolute intensity). 1, pyroglutamic acid; 2, glyceric acid; 3, lactic acid; 4, acetic acid; 5, propionic acid; 6, iso-butyric acid; 7, butyric acid; 8, 2-methylbutyric acid; 9, iso-valeric acid; 10, 2-ethylbutyric acid; 11, valeric acid; 12, 3,3-dimethylbutyric acid; 13, 2-methylvaleric acid; 14, 3-methylvaleric acid; 15, 4-methylvaleric acid; 16, hexanoic acid; 17, phenylacetic acid; 18, succinic acid; 19, malic acid; 20, malonic acid; 21, 10-hydroxydecanoic acid; 22, benzoic acid; 23, fumaric acid; 24, 3-phenylpropionic acid; 25, 4-hydroxyphenylacetic acid; 26, 4-hydroxybenzoic acid; 27, caprylic acid; 28, 3-hydroxyphenylacetic acid; 29, 3-(3-hydroxyphenyl)propionic acid

AIC-HR-MS was able to detect all compounds except medium- and long-chain fatty acids with ten or more carbon atoms and without polar substituents, such as hydroxy-carboxylic acids. Chromatograms of CAs in standard solution and in a fecal extract are shown in Fig. 4. Isomeric SCFAs could be separated, although baseline separation could not be achieved for every analyte pair. Hexanoic acid and caprylic acid produced highly tailing peaks due to lipophilic interactions with the stationary phase. Carryover of some CAs into blanks was also observed in AIC-HR-MS. Lactic acid and dicarboxylic acids (succinic acid, malic acid, malonic acids, 3-hydroxyglutaric acid) showed greater carryover than benzoic acid, pyroglutamic acid, and caprylic acid.

### Extraction of feces and ruminal fluid

Both Han et al. [11] and Chan et al. [10] extracted human feces with ACN/water (50/50, *v/v*). As we were interested also in more lipophilic medium- and long-chain fatty acids, we compared the peak areas of derivatized CAs extracted from feces and ruminal fluid with ACN/water in the ratios 80/20 and 50/50 (*v*/*v*). For the internal standards ^13^C-labeled acetic, propionic, and butyric acid, the peak areas after extraction with ACN/water (80/20, *v*/*v*) were 117–131% of those obtained after extraction with equal portions of ACN and water. Native CAs from feces and ruminal fluid yielded similar or higher peak areas after extraction with ACN/water (80/20, *v*/*v*). The greatest differences were observed for long-chain fatty acids where ACN/water (50/50, *v*/*v*) yielded only 52–69% extraction compared to ACN/water (80/20, *v*/*v*), based on the peak areas. For that reason, ACN/water (80/20, *v*/*v*) was chosen as extraction solvent in all further experiments.

### Method validation

Limits of detection (LODs), limits of quantification (LOQs), and the linear range of the derivatizable CAs in standard solutions are listed in Table 1. For most compounds, 3-NPH derivatives had similar or lower LODs and LOQs and a lower upper limit of quantification than aniline derivatives. The highest LODs and LOQs were obtained by AIC-HR-MS, where non-derivatized compounds were measured that generally ionize worse than derivatized compounds. In addition, the comparison is biased because a high-resolution mass spectrometer with different ion source was used instead of a triple quadrupole instrument. LODs and LOQs in feces and ruminal fluid are given in Online Resource Table S7.

**Table 1:** Limits of detection (LOD), limits of quantification (LOQ), and linear range (upper limit of quantification, LLOQ) of the investigated carboxylic acids (ng/mL in measurement solution). *CO* could not be determined due to carryover; *n.d.* not detected

	Aniline derivatization			3-NPH derivatization			AIC-HR-MS		
	LOD	LOQ	ULOQ	LOD	LOQ	ULOQ	LOD	LOQ	ULOQ
Acetic acid	2.9	29	7710	8.6	29	2286	100	333	40000
Propionic acid	2.9	8.6	4290	0.3	2.9	286	20	67	15000
Butyric acid	2.9	29	7710	0.9	8.7	286	9.0	30	15000
Iso-Butyric acid	4.0	12	7710	8.7	29	2286	12	40	15000
Lactic acid	CO	86	11400	CO	86	857	CO	600	15000
2-Methylbutyric acid	2.9	8.6	7710	0.9	2.9	2286	10	33	15000
Valeric acid	0.9	8.6	2860	0.9	8.7	286	10	33	15000
Iso-Valeric acid	0.9	8.6	2860	0.9	8.7	286	10	33	15000
3,3-Dimethylbutyric acid	2.9	8.6	7710	2.9	8.6	286	5.0	16	15000
Glyceric acid	2.9	8.6	286	2.9	8.6	286	21	100	1500
Hexanoic acid	0.9	8.6	7710	0.3	8.7	286	1.0	4.0	15000
2-Ethylbutyric acid	2.9	8.6	7710	0.3	8.7	286	5.0	16	15000
3-Methylvaleric acid	2.9	8.6	7710	2.9	8.6	857	4.0	15	15000
2-Methylvaleric acid	2.9	8.6	11400	0.6	2.9	857	4.0	15	15000
4-Methylvaleric acid	0.9	2.9	7710	0.9	2.9	2286	5.0	16	15000
Benzoic acid	n.d.	n.d.	n.d.	2.9	29	286	1.0	10	15000
Pyroglutamic acid	8.6	29	2860	0.9	8.6	2286	CO	10	15000
Phenylacetic acid	0.9	8.6	2860	0.6	2.9	286	30	100	15000
4-Hydroxybenzoic acid	29	86	2860	0.9	2.9	286	10	33	15000
Caprylic acid	CO	29	2860	CO	8.7	286	50	167	15000
3-Phenylpropionic acid	0.9	8.6	2860	0.3	1.0	286	5.0	17	15000
4-Hydroxyphenylacetic acid	0.9	2.9	2860	0.9	2.9	286	12	60	15000
3-Hydroxyphenylacetic acid	2.9	8.6	2860	2.0	7.0	2286	50	160	15000
3-(3-Hydroxyphenyl)propionic acid	1.0	2.9	2860	0.9	2.9	286	18	60	15000
Decanoic acid	CO	8.6	857	2.9	8.7	286	n.d.	n.d.	n.d.
Undecanoic acid	CO	8.6	857	0.9	8.7	286	n.d.	n.d.	n.d.
10-Hydroxydecanoic acid	2.0	6.0	2860	0.9	2.9	286	30	100	15000
Lauric acid	CO	8.6	857	2.9	8.7	86	n.d.	n.d.	n.d.
Myristic acid	CO	8.6	857	0.9	2.9	86	n.d.	n.d.	n.d.
Malonic acid (2 × D, 1 charge)	5.0	15	7710	2.9	8.7	857	10	33	3000
Pentadecanoic acid	CO	8.6	86	2.9	8.6	86	n.d.	n.d.	n.d.
Fumaric acid (2 × D, 1 charge)	5.0	15	7710	2.9	8.7	857	10	33	3000
Succinic acid (2 × D, 1 charge)	2.0	6.0	857	2.9	8.7	286	10	33	3000
Palmitic acid	CO	86	857	2.9	29	286	n.d.	n.d.	n.d.
Malic acid (2 × D, 1 charge)	2.9	8.6	7710	2.9	8.7	857	10	33	3000
Linoleic acid	CO	29	857	8.6	29	857	n.d.	n.d.	n.d.
Oleic acid	CO	29	857	8.6	29	286	n.d.	n.d.	n.d.
3-Hydroxyglutaric acid (2 × D, 1 charge)	29	86	2860	12	40	2286	30	100	10000
Stearic acid	CO	29	286	8.6	29	286	n.d.	n.d.	n.d.

For all three tested methods, the repeatability for the internal standards (^13^C-labeled acetic, propionic, and butyric acid, all added prior to extraction), expressed by the relative standard deviation (RSD) of quadruplicate sample preparation and single measurement of each feces and ruminal fluid extract, was below 10% and mostly between 2 and 5%. For the major SCFAs acetic, propionic, butyric, and valeric acid, the repeatability of sample preparation and measurement was between 2.5 and 11% for both derivatization methods and the AIC-HR-MS approach. Online Resource Table S8 gives the repeatability for all compounds detected in feces and ruminal fluid by the investigated methods. RSDs above 15% were observed mainly for compounds near the LOQ of the respective method and for compounds that were not baseline separated.

Apparent recoveries (including recovery of extraction, derivatization yield, and mass spectrometric matrix effects) were determined only for acetic acid, propionic acid, and butyric acid whose ^13^C-labeled forms were commercially available and affordable. *R*_*A*_s were assessed only by means of the isotopically labeled internal standards because the determination of *R*_*A*_s required spiking of compounds prior to extraction. Standard addition was ruled out due to the high concentrations of SCFAs in cow feces and ruminal fluid and as the spiking solution must not be evaporated to avoid analyte losses. *R*_*A*_s of ^13^C-labeled acetic, propionic, and butyric acid were between 45 and 57% for aniline derivatization and between 89 and 96% for 3-NPH derivatization (Table 2). In ruminal fluid, *R*_*A*_s were between 30 and 45% for aniline derivatives and between 101 and 110% for 3-NPH derivatives. *R*_*A*_s of ^13^C-labeled internal standards measured by AIC-HR-MS were between 94 and 120% in feces and between 108 and 135% in ruminal fluid (Table 2). To further investigate the reason for the poor *R*_*A*_s after derivatization with aniline, *R*_*A*_s were also calculated for each individual spiking level of the standard addition samples. Whereas *R*_*A*_s were similar over the whole spiking range (0.5–3 × of the analyte concentration determined in a pre-experiment) for derivatization with 3-NPH and for the AIC-HR-MS method, *R*_*A*_s of all three ^13^C-labeled standards increased with increasing spiking level for derivatization with aniline both in feces and ruminal fluid extracts. Average *R*_*A*_s of ^13^C-acetic acid, ^13^C-propionic acid, and ^13^C-butyric acid linearly increased from 55% in the unspiked feces extract to 85% at the threefold spiking level and from 37% in the unspiked ruminal fluid extract to 83% at the highest spiking level in ruminal fluid extract. Closer investigation of the peak areas of the ^13^C-labeled internal standards revealed that, although peak areas should be similar throughout the spiking levels, peak areas increased with increasing spiking concentration after aniline derivatization. Peak areas of aniline-derivatized internal standards were approximately doubled at spiking level 0.5 compared to the unspiked samples both in standard solutions and in sample extracts. At spiking levels 2, 2.5, and 3, peak areas were 1.6–3.2 times higher than in the unspiked standard solutions and 3.4–4.4 times higher than in the unspiked feces and ruminal fluid extracts. This higher increase in sample extracts than in standard solutions is the reason for greater *R*_*A*_s at higher spiking levels. However, it does not explain the low *R*_*A*_s of the internal standards in the unspiked samples. In strong contrast, upon derivatization with 3-NPH, peak areas of ^13^C-labeled internal standards were similar across all spiking levels.

**Table 2:** Apparent recoveries ± standard deviation of ^13^C-labeled internal standards added prior to sample work-up (*n* = 4).

		^13^C-Acetic acid	^13^C-Propionic acid	^13^C-Butyric acid
Aniline derivatization	Feces	63 ± 2	52 ± 2	50 ± 2
	Ruminal fluid	45 ± 3	34 ± 3	31 ± 3
3-NPH derivatization	Feces	89 ± 3	96 ± 4	94 ± 1
	Ruminal fluid	101 ± 4	110 ± 6	109 ± 5
AIC-HR-MS	Feces	120 ± 5	98 ± 2	94 ± 2
	Ruminal fluid	135 ± 2	113 ± 1	108 ± 2

As the extraction step was the same for the two derivatization methods and the AIC-HR-MS method, the reason for the low *R*_*A*_s after derivatization with aniline was either poor derivatization yield or mass spectrometric signal suppression. Mass spectrometric matrix effects were between 75 and 124% and mostly around 100% for all compounds in feces and ruminal fluid extracts derivatized by aniline and 3-NPH, respectively. Therefore, poorer derivatization yield in matrix compared to pure solvent solutions remained as the only reason for low *R*_*A*_s of the added internal standards after aniline derivatization in both cow matrices. However, the derivatization yields in matrix relative to the derivatization yield in pure solvent standards were around 100% for both aniline and 3-NPH derivatization if assessed by comparison of the slopes of the calibration curves (Table 3). For both derivatization methods, missing values were due to bad calibration curves, mostly resulting from low spiked analyte concentrations or due to the absence of analytes in the spiking solutions as in the case of fatty acids in ruminal fluid. If the derivatization yield was assessed at the individual spiking levels by quantifying spiked sample extracts on the basis of external calibration curves (see “Quantification of CAs in animal samples”), subtracting the analyte concentrations in the unspiked samples and comparing the measured concentrations to the spiked concentrations (values not shown), the average recovery of the spiked concentrations was between 80 and 130% for most analytes. The only exceptions were pyroglutamic acid (58%) that showed a bad calibration curve and iso-butyric acid (254%) that was affected by the partly co-eluting butyric acid. The good derivatization yields were surprising as they did not explain the low *R*_*A*_s of ^13^C-labeled internal standards. In a next step, CAs were quantified in the unspiked samples to investigate whether both derivatization methods and AIC-HR-MS yield similar results despite different *R*_*A*_s of labeled internal standards.

**Table 3:** Derivatization yield in matrix ± standard deviation (%) determined by standard addition method (*n* = 2). *n.a.* not assessed

	Aniline derivatization		3-NPH derivatization	
	Feces	Ruminal fluid	Feces	Ruminal fluid
Acetic acid	91 ± 3	91 ± 1	90 ± 4	92 ± 6
Propionic acid	105 ± 2	104 ± 3	94 ± 0	85 ± 1
Butyric acid	101 ± 1	96 ± 14	98 ± 3	94 ± 1
Valeric acid	97 ± 1	106 ± 2	97 ± 1	103 ± 6
Hexanoic acid	102 ± 1	104 ± 0	n.a.	100 ± 6
Pyroglutamic acid	114 ± 7	82 ± 2	98 ± 7	n.a.
Phenylacetic acid	110 ± 0	135 ± 2	100 ± 5	118 ± 10
3-Phenylpropionic acid	107 ± 0	110 ± 0	97 ± 0	102 ± 5
4-Hydroxyphenylacetic acid	103 ± 1	102 ± 6	99 ± 0	133 ± 17
3-(3-Hydroxyphenyl)propionic acid	108 ± 3	102 ± 6	103 ± 3	143 ± 9
Iso-Butyric acid	104 ± 4	212 ± 10	96 ± 2	129 ± 5
2-Methylbutyric acid	96 ± 2	103 ± 0	99 ± 1	102 ± 3
4-Methylvaleric acid	101 ± 4	96 ± 1	98 ± 7	n.a.
3-Hydroxyphenylacetic acid	103 ± 9	103 ± 6	100 ± 2	n.a.
Iso-Valeric acid	114 ± 2	141 ± 4	94 ± 0	101 ± 12
Myristic acid	100 ± 3	n.a.	74 ± 3	n.a.
Palmitic acid	153 ± 8	n.a.	n.a.	n.a.
Linoleic acid	119 ± 3	n.a.	92 ± 8	n.a.
Oleic acid	100 ± 8	n.a.	99 ± 3	n.a.

### Quantification of carboxylic acids in animal samples

Concentrations of CAs in the feces and ruminal fluid samples used for validation are presented in Tables 4 and 5. The major CAs were the SCFAs acetic, propionic, and butyric acid, occurring in the high mg/kg or mg/L range. Concentrations calculated on the basis of pure solvent calibration curves and by standard addition method were similar, indicating good consistency of the quantification approaches. For all analytes except pyroglutamic acid, derivatization with 3-NPH and AIC-HR-MS yielded similar concentrations. However, derivatization with aniline yielded on average only around 20% of the concentrations obtained by the other methods. For that reason, some analytes could only be reported as “traces” although they could be quantified after derivatization with 3-NPH. Correction of the concentrations obtained by external calibration by the *R*_*A*_s of ^13^C-acetic acid, ^13^C-propionic acid, and ^13^C-butyric acid increased the values to approximately 50% of the concentrations obtained by derivatization with 3-NPH and AIC-HR-MS. Still, the other 50% remain untraceable. To more closely investigate this inconsistency upon aniline derivatization, also sample extracts spiked in the course of validation were quantified on the basis of external calibration curves, and derivatization recoveries were calculated at each spiking level (see above). Average recoveries of the spiked concentrations between 80 and 130% for most analytes indicated that derivatization with aniline worked fine for compounds if they were spiked to the sample extract but not if they were naturally occurring. This finding warranted further investigation of the derivatization conditions.

**Table 4:** Average concentrations (mg/kg wet basis, *n* = 2) in feces used for validation determined by external calibration curve (Ex cal) and by standard addition method (SA). Phenylacetic acid, 4-methylvaleric acid, and 3-hydroxyphenylacetic acid occurred in traces, whereas palmitic acid was below the LOD in all methods. *tr* traces; *R*_*A*_* corr* correction by the apparent recovery of the ^13^C-labeled internal standards; *n.d.* not detected. RSDs of duplicate work-up and measurement are given in Online Resource Table S9

	Aniline derivatization		3-NPH Derivatization		AIC-HR-MS	
	Ex cal (Ex cal + *R*_*A*_ corr)	SA	Ex cal	SA	Ex cal	SA
Acetic acid	588 (934)	591	2188	2536	1950	1894
Propionic acid	114 (219)	113	557	602	534	400
Butyric acid	56 (111)	57	269	301	262	242
Valeric acid	4.8 (8.7)	5.4	55	49	56	55
Hexanoic acid	tr	tr	tr	tr	2.1	4.5
Pyroglutamic acid	9.0 (16)	3.3	72	63	6.4	6.7
3-Phenylpropionic acid	4.2 (7.7)	3.4	20	16	18	20
4-Hydroxyphenylacetic acid	tr	tr	3.6	2.2	tr	tr
3-(3-Hydroxyphenyl)propionic acid	7.5 (14)	8.3	35	25	35	41
Iso-Butyric acid	6.4 (12)	4.4	42	36	41	39
2-Methylbutyric acid	tr	tr	16	13	15	13
Iso-Valeric acid	tr	tr	12	13	13	12
Myristic acid	3.7 (6.7)	2.6	25	39	n.d.	n.d.
Linoleic acid	27 (49)	26	162	166	n.d.	n.d.
Oleic acid	13 (23)	13	78	80	n.d.	n.d.

**Table 5:** Average concentrations (mg/L wet basis, *n* = 2) in ruminal fluid used for validation determined by external calibration curve (Ex cal) and by standard addition method (SA). Phenylacetic acid, 4-methylvaleric acid, and 3-hydroxyphenylacetic acid occurred in traces, whereas 4-hydroxyphenylacetic acid, 3-(3-hydroxyphenyl)propionic acid, 4-methylvaleric acid, 3-hydroxyphenylacetic acid, and all investigated long-chain fatty acids were below the LOD in all methods. *tr* traces; *R*_*A*_* corr* correction by the apparent recovery of the ^13^C-labeled internal standards; *n.d.* not detected. Analytes without ^13^C-labeled internal standard were corrected by the average *R*_*A*_ of ^13^C-acetic acid, ^13^C-propionic acid, and ^13^C-butyric acid. RSDs of duplicate work-up and measurement are given in Online Resource Table S10

	Aniline derivatization		3-NPH derivatization		AIC-HR-MS	
	Ex cal (Ex cal + *R*_*A*_ corr)	SA	Ex cal	SA	Ex cal	SA
Acetic acid	877 (1949)	732	3906	4245	3890	3321
Propionic acid	220 (646)	223	1187	1112	1189	956
Butyric acid	197 (634)	204	1018	1042	1026	959
Valeric acid	17 (47)	18	96	104	98	101
Hexanoic acid	8.7 (24)	7.8	48	60	48	53
Pyroglutamic acid	n.d.	n.d.	n.d.	n.d.	tr	5.2
3-Phenylpropionic acid	16 (44)	17	73	76	72	78
Iso-Butyric acid	6.4 (17)	7.2	42	35	74	66
2-Methylbutyric acid	tr	tr	28	28	22	29
Iso-Valeric acid	4.9 (13)	3.1	35	46	46	39

Chan and co-workers [10] quantified SCFAs in infant stool by preparing ^13^C-labeled calibration standards and spiking these standards into concentrated and diluted stool extracts. However, this approach corrects only for mass spectrometric matrix effects, but not for any differences in derivatization yield upon derivatization in solvent and in matrix solution.

The applicability of the two derivatization methods and AIC-HR-MS to animal matrices was tested by quantifying CAs in feces and ruminal fluid of Holstein non-lactating rumen-cannulated cows that were gradually exposed to a high-grain diet (Tables 6 and 7). Again, similar concentrations were obtained by 3-NPH derivatization and AIC-HR-MS with the exception of iso-butyric acid which showed partial co-elution with the more concentrated butyric acid in both methods. Aniline derivatization yielded only about 20% of the concentrations determined by the other methods. Concerning the metabolite pattern, the SCFAs acetic, propionic, and butyric acid were the major metabolites in feces and ruminal fluid. Valeric, iso-butyric, iso-valeric, and 2-methylbutyric acid also occurred in both matrices, whereas hexanoic acid could only be detected in ruminal fluid. 3-Phenylpropionic acid, phenylacetic acid, and pyroglutamic acid could be quantified in feces and ruminal fluid, and 4-hydroxyphenylacetic acid was above the LOQ in feces. Long-chain fatty acids (oleic and myristic acid) were detected only in feces samples.

**Table 6:** Concentrations of carboxylic acids (mg/kg wet basis) in feces of non-lactating cows fed high-concentrate diet. Hexanoic acid and 4-methylvaleric acid were below the respective LODs in all methods. *tr* traces; *n.d.* not detected

	Feces (mg/kg)								
	Aniline derivatization			3-NPH derivatization			AIC-HR-MS		
	W 1	W 2	W 4	W 1	W 2	W 4	W 1	W 2	W 4
Acetic acid	422	701	601	1496	2557	1821	1279	2191	1828
Propionic acid	68	138	114	332	663	536	328	621	516
Butyric acid	28	56	55	146	272	269	128	252	249
Valeric acid	8.0	12	11	50	67	58	51	68	60
Pyroglutamic acid	tr	tr	tr	15	14	20	tr	tr	tr
Phenylacetic acid	tr	tr	tr	15	5.0	6.0	tr	n.d.	n.d.
3-Phenylpropionic acid	5.1	5.0	4.6	25	23	22	25	22	20
4-Hydroxyphenylacetic acid	tr	1.6	tr	8.0	8.0	4.6	tr	tr	tr
3-(3-Hydroxyphenyl)propionic acid	tr	1.5	tr	tr	8.3	tr	n.d.	n.d.	n.d.
Iso-Butyric acid	7.1	7.0	7.1	74	61	71	54	50	57
2-Methylbutyric acid	n.d.	n.d.	n.d.	26	19	21	23	15	18
3-Hydroxyphenylacetic acid	n.d.	n.d.	n.d.	tr	tr	tr	n.d.	n.d.	n.d.
Iso-Valeric acid	tr	tr	tr	29	17	22	29	18	21
Myristic acid	tr	tr	4.3	36	21	33	n.d.	n.d.	n.d.
Palmitic acid	n.d.	n.d.	n.d.	tr	tr	tr	n.d.	n.d.	n.d.
Linoleic acid	n.d.	n.d.	n.d.	tr	tr	tr	n.d.	n.d.	n.d.
Oleic acid	tr	tr	tr	48	65	90	n.d.	n.d.	n.d.

**Table 7:** Concentrations of carboxylic acids (mg/L wet basis) in ruminal fluid of non-lactating cows fed high-concentrate diet. 4-Hydroxyphenylacetic acid, 3-hydroxyphenylacetic acid, 3-(3-hydroxyphenyl)propionic acid, and 4-methylvaleric acid were below the respective LODs in all methods. *tr* traces; *n.d.* not detected

	Ruminal fluid (mg/L)								
	Aniline derivatization			3-NPH derivatization			AIC-HR-MS		
	W 1	W 2	W 4	W 1	W 2	W 4	W 1	W 2	W 4
Acetic acid	572	961	1130	3898	4450	4552	3711	4438	4818
Propionic acid	124	260	341	1224	1498	1751	1220	1536	1918
Butyric acid	74	250	270	725	1418	1329	706	1498	1388
Valeric acid	13	33	38	148	202	199	150	212	221
Hexanoic acid	9.0	20	15	101	114	74	109	123	82
Pyroglutamic acid	n.d.	n.d.	n.d.	18	76	102	tr	10	10
Phenylacetic acid	tr	tr	6.5	21	16	29	n.d.	n.d.	n.d.
3-Phenylpropionic acid	13	18	20	116	93	88	118	95	92
Iso-Butyric acid	tr	tr	10	88	66	70	183	147	151
2-Methylbutyric acid	tr	7.1	8.2	101	104	96	115	107	107
Iso-Valeric acid	tr	5.9	8.2	72	58	72	62	52	69

The influence of the diet on the CA profile was seen in feces and in ruminal fluid samples. In feces, the SCFAs acetic acid, propionic acid, butyric acid, and valeric acid were increased in the first and third week of concentrate feeding compared to forage feeding, indicating acidification in the GI tract due to an excess of easily fermentable carbohydrates. In ruminal fluid, acetic, propionic, butyric, valeric, and pyroglutamic acids were increased due to high-grain feeding.

### Testing parameters for derivatization of carboxylic acids with aniline

We tested if the rather unexpected low derivatization yield with aniline in biological matrices could be explained or potentially rectified by changing the derivatization conditions. Increase of the derivatization temperature to 40 °C significantly increased the peak area of acetic acid in standard solution (136% compared to derivatization at 4 °C) but slightly decreased the peak areas of the other investigated CAs (73–95% compared to derivatization at 4 °C), with significant decreases only for iso-butyric and iso-valeric acid. Interestingly, derivatization with threefold higher reagent concentration of aniline and EDC significantly decreased the peak areas of all investigated CAs in standard solution, with values between 78 and 85% compared to regular derivatization.

**Fig. 5:**
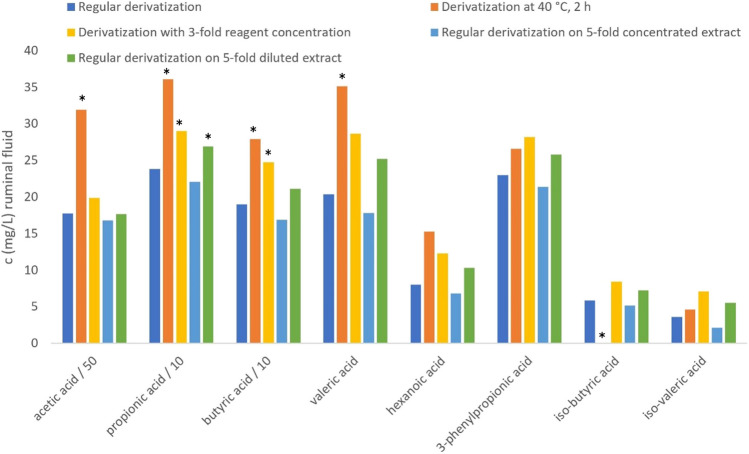
Concentrations of carboxylic acids (mg/L) in ruminal fluid determined by different derivatization conditions with aniline as derivatization agent. *, statistically significant compared to regular derivatization (α = 0.05)

Contrary to the results in pure standard solutions, CAs in ruminal fluid derivatized at 40 °C were mostly significantly increased (α = 0.05) compared to CAs derivatized at 4 °C (Fig. 5). Only iso-butyric acid was below the LOD after derivatization at 40 °C. Derivatization with the threefold concentration of aniline and EDC significantly increased the peak areas of propionic and butyric acid and increased the peak areas of all other CAs compared to regular derivatization. However, regular derivatization of a fivefold more concentrated ruminal fluid extract yielded similar results as derivatization of a normal extract. Derivatization of a five times more diluted extract than the normal extract yielded significantly higher peak areas of propionic acid and slightly increased peak areas of the other CAs.

These findings indicate that derivatization with aniline is little rugged as changes in parameters like derivatization temperature and reagent to analyte ratio cause significant differences in peak areas. These differences vary for derivatization in standard solution and sample extract. For that reason, derivatization with aniline is not recommended for quantitative analysis of CAs in animal samples.

### Comparison of the three methods for determination of carboxylic acids in animal samples

In terms of ease of performance, AIC-HR-MS is the simplest method as extracts can be measured directly after dilution. Derivatization with 3-NPH is the faster and more convenient of the two derivatization approaches. The derivatization step lasts 30 min, and quenching is not required. With two incubation periods (derivatization and quenching) for 2 h each, derivatization with aniline is the most complex method.

Regarding the number of determinable compounds, 38 out of 39 tested compounds could be derivatized with aniline, and all 39 compounds could be derivatized with 3-NPH. AIC-HR-MS detected the least number of compounds (29) because medium- and long-chain fatty acids with ≥ 10 carbon atoms could not be eluted from the column. However, medium- and long-chain fatty acids were problematic analytes also in the derivatization methods where they showed carryover especially after aniline derivatization. In addition, calibration curves of derivatized medium- and long-chain fatty acids tended to have lower correlation coefficients both after aniline and 3-NPH derivatization.

The derivatization efficiency proved to be the crucial point in distinguishing the derivatization methods. Whereas derivatization with 3-NPH was complete for all investigated compounds both in solvent solutions and in matrix extract, derivatization with aniline resulted in derivatization efficiencies between 20 and 100%. In addition, derivatization efficiencies differed in solvent solutions and matrix extracts. Changes in derivatization parameters like temperature and reagent concentrations also impacted the derivatization efficiencies. As a consequence of variable derivatization yields, average *R*_*A*_s of ^13^C-labeled internal standards added prior to sample preparation were 37% in ruminal fluid and 55% in feces after aniline derivatization, whereas derivatization with 3-NPH and measurement by AIC-HR-MS resulted in *R*_*A*_s close to 100%. LODs and LOQs were similar for both derivatization methods, with on average slightly lower values for derivatization with 3-NPH, while AIC-HR-MS was the least sensitive of the three tested methods with the highest linear range.

Concentrations of CAs in feces and ruminal fluid determined by derivatization with 3-NPH and AIC-HR-MS were similar, whereas concentrations obtained by derivatization with aniline were on average only 20% of those determined by the other methods. This finding and the variable derivatization efficiency indicate that derivatization with aniline is not reliable for CA quantification. In contrast, derivatization with 3-NPH passed all validation experiments and quantification results matched those obtained by AIC-HR-MS. In addition, 3-NHP has been successfully used for derivatization of CAs in other studies [17, 20]. Consequently, of the two approaches tested, derivatization with 3-NPH is preferable for metabolome profiling.

## Conclusion

Both tested derivatization methods and the AIC-HR-MS method were suitable for qualitative determination of structurally diverse CAs in animal matrices. Derivatization with 3-NPH performed well in all validation experiments and yielded similar CA concentrations as the complementary reference method AIC-HR-MS. In contrast, derivatization with aniline had variable derivatization efficiencies, lower apparent recoveries, and yielded on average five times lower CA concentrations in feces and ruminal fluid samples than the other two methods. Variation of derivatization conditions with aniline (increase of reaction temperature, increase of reagent concentration) and correction by the apparent recoveries increased the CA concentrations in samples by a factor of 2 to 3 but could not account for a factor of 5 compared to derivatization with 3-NPH and AIC-HR-MS measurement. Therefore, derivatization with 3-NPH is preferred over derivatization with aniline for quantitative CA determination in animal matrices in the course of metabolome profiling.

### Supplementary Information

Below is the link to the electronic supplementary material.

Supplementary file1 (DOCX 72.2 KB)
