# Supplementary material for: Comparison of LC-MS-based methods for the determination of carboxylic acids in animal matrices
Source: Anal Bioanal Chem. 2024 Jan 5;416(5):1199–215. doi: 10.1007/s00216-023-05113-8 (PMC10850028; doi:10.1007/s00216-023-05113-8)
Supplement: Supplementary file 1 — Supplementary file1 (DOCX 72.2 KB) [file 216_2023_5113_MOESM1_ESM.docx]

Electronic Supplementary Information to „**Comparison of LC-MS based methods for the determination of carboxylic acids in animal matrices**”

Analytical and Bioanalytical Chemistry

Heidi E. Schwartz‑Zimmermann^1,2^ · Manuel Hündler^1,2,3^ · Nicole Reiterer^1,2,4^ · Sara Ricci^2,5^ ·

Raul Rivera‑Chacon^2,5^ · Ezequias Castillo‑Lopez^2,5^ · Qendrim Zebeli^2,5^ · Franz Berthiller^1,2^

^1^ Department of Agrobiotechnology, IFA‑Tulln, Institute of Bioanalytics and Agro-Metabolomics,

University of Natural Resources and Life Sciences, Vienna (BOKU), Tulln, Austria

^2^ Christian Doppler Laboratory for Innovative Gut Health Concepts of Livestock, Vienna, Austria

^3^ Pfizer Austria, Orth an der Donau, Austria

^4^ Marinomed Biotech AG, Korneuburg, Austria

^5^ Department for Farm Animals and Veterinary Public Health, Institute of Animal Nutrition and Functional Plant Compounds, University of Veterinary Medicine Vienna, Vienna, Austria

*Corresponding author: [heidi.schwartz@boku.ac.at](mailto:heidi.schwartz@boku.ac.at)

**Sources of reference standards**

Carboxylic acid reference standards were purchased from Sigma-Aldrich (2-methylbutyric acid, 2-ethylbutyric acid, 3-hydroxyglutaric acid, 3-phenylpropionic acid, 10-hydroxydecanoic acid, acetic acid, decanoic acid, fumaric acid, glyceric acid, lactic acid, malic acid, oleic acid, palmitic acid, propionic acid, pyroglutamic acid, stearic acid, succinic acid, undecanoic acid, and the internal standards ^13^C_2_-acetic acid, ^13^C_4_-butyric acid, ^13^C_3_-propionic acid), VWR (3-methylvaleric acid, 3,3-dimethylbutyric acid, 3-(3-hydroxyphenyl)propionic acid, 4-methylvaleric acid, benzoic acid, iso-butyric acid, malonic acid, phenylacetic acid), Merck (4-hydroxybenzoic acid, 3-hydroxyphenylacetic acid, 4-hydroxyphenylacetic acid, caprylic acid, lauric acid, pentadecanoic acid), TCI-Germany, Eschborn, Germany (2-methylvaleric acid, butyric acid, hexanoic acid, iso-valeric acid, valeric acid), Acros Organics (myristic acid) and Extrasynthese, Genay, France (linoleic acid).

**Derivatization conditions**

Chan and co-workers [1] originally derivatized a 100 µL aliquot of sample extract (ratio of extraction solvent to wet human stool sample was 2 µL to 1 mg) using a final concentration of 10 mM aniline and 5 mM EDC. This corresponds to 1 µmol aniline absolute. According to Chan et al., the sum concentration of short-chain fatty acids (SCFAs) in human feces is within the low ng/g range. In contrast, ruminant feces contains SCFA levels in the medium µmol/g range. Considering that we use aliquots of extracts containing 1.5 mg of feces and 1.8 µL of ruminal fluid for derivatization, the total amount of SCFAs for derivatization is between 0.1 and 1 µmol. Based on previous experiments showing the influence of the reagent concentration on the derivatization efficiency, we used 5 µmol aniline in the derivatization solution, and likewise 5 µmol of EDC which is consumed stoichiometrically during derivatization.

**Preparation of mixed stock solutions for establishing calibration curves and for validation experiments**

To determine the retention times, limits of detection (LOD) and quantification (LOQ) as well as the linearity of carboxylic acids, compounds were grouped into mixes containing 40 mg/L of each compound (unless stated otherwise) according to their molecular mass. The following mixes were prepared:

**Table S1**: Mixes for determination of retention times, LOD, LOQ

| Mix 1 | Mix 2 | Mix 3 |
| --- | --- | --- |
| acetic acid | iso-butyric acid | 2-methylvaleric acid |
| propionic acid | 2-methylbutyric acid | myristic acid |
| butyric acid | 4-methylvaleric acid | pentadecanoic acid |
| lactic acid | 3-hydroxyphenylacetic acid | palmitic acid |
| valeric acid | 3-hydroxyglutaric acid | linoleic acid |
| hexanoic acid |  | oleic acid |
| benzoic acid |  | stearic acid |
| pyroglutamic acid |  |  |
| phenylacetic acid |  |  |
| 4-hydroxybenzoic acid |  |  |
| caprylic acid |  |  |
| 3-phenylpropionic acid |  |  |
| 4-hydroxyphenylacetic acid |  |  |
| 3-(3-hydroxyphenyl)propionic acid |  |  |
| decanoic acid |  |  |
| undecanoic acid |  |  |
| 10-hydroxydecanoic acid |  |  |
| lauric acid |  |  |
| succinic acid |  |  |
| malic acid |  |  |
| malonic acid |  |  |

In addition, single compound solutions of 2-ethylbutyric acid, iso-valeric acid, 3,3-dimethylbutyric acid, 3-methylvaleric acid and fumaric acid were prepared at 20 mg/L, and a stock solution of glyceric acid was prepared at 10 mg/L.

**Production of ^13^C-labeled injection standards**

**Table S2**: Preparation of injection standard mixes for feces and ruminal fluid samples by derivatization of a standard mix with ^13^C_6_-anilin or ^13^C_6_-3-NPH, respectively. 5 µL aliquots of the derivatized injection standard mixes were added to 145 µL of derivatized standard or sample solution, resulting in the concentrations in measurement solution given in the right column.

| **Analytes in standard mix** | **conc in mix for derivatization (mg/L)** | **conc in derivatized mix (mg/L)** | **conc in measurement solution (mg/L)** |
| --- | --- | --- | --- |
| Feces | | | |
| acetic acid | 163 | 6.0 | 0.200 |
| butyric acid | 54.4 | 2.0 | 0.067 |
| propionic acid | 54.4 | 2.0 | 0.067 |
| 3-(3-hydroxyphenyl)propionic acid | 43.6 | 1.6 | 0.053 |
| linoleic acid | 43.6 | 1.6 | 0.053 |
| myristic acid | 43.6 | 1.6 | 0.053 |
| oleic acid | 43.6 | 1.6 | 0.053 |
| palmitic acid | 43.6 | 1.6 | 0.053 |
| valeric acid | 43.6 | 1.6 | 0.053 |
| 2-methylbutyric acid | 21.8 | 0.8 | 0.027 |
| 3-phenylpropionic acid | 21.8 | 0.8 | 0.027 |
| 4-hydroxyphenylacetic acid | 21.8 | 0.8 | 0.027 |
| 3-hydroxyphenylacetic acid | 21.8 | 0.8 | 0.027 |
| 4-methylvaleric acid | 21.8 | 0.8 | 0.027 |
| iso-butyric acid | 21.8 | 0.8 | 0.027 |
| iso-valeric acid | 21.8 | 0.8 | 0.027 |
| phenylacetic acid | 21.8 | 0.8 | 0.027 |
| pyroglutamic acid | 21.8 | 0.8 | 0.027 |
| Ruminal fluid | | | |
| acetic acid | 163 | 6.0 | 0.200 |
| butyric acid | 54.4 | 2.0 | 0.067 |
| propionic acid | 54.4 | 2.0 | 0.067 |
| 3-phenylpropionic acid | 43.6 | 1.6 | 0.053 |
| hexanoic acid | 43.6 | 1.6 | 0.053 |
| valeric acid | 43.6 | 1.6 | 0.053 |
| 2-methylbutyric acid | 21.8 | 0.8 | 0.027 |
| 4-hydroxyphenylacetic acid | 21.8 | 0.8 | 0.027 |
| 3-hydroxyphenylacetic acid | 21.8 | 0.8 | 0.027 |
| iso-butyric acid | 21.8 | 0.8 | 0.027 |
| iso-valeric acid | 21.8 | 0.8 | 0.027 |
| phenylacetic acid | 21.8 | 0.8 | 0.027 |
| pyroglutamic acid | 21.8 | 0.8 | 0.027 |

**Table S3**: Selected reaction monitoring (SRM) parameters for determination of 3-NPH derivatives (negative ionization mode). The declustering potential was -70 eV for all compounds. CE: collision energy; t_R_: retention time; 2 x D: derivatized on 2 functional groups; n.d.: not detected; InjS-^13^C: injection standard prepared in-house with ^13^C-aniline; ^13^C_2_-acetic acid, ^13^C_3_-propionic acid and ^13^C_4_-butyric acid: fully labeled internal standards derivatized with native 3-NPH.

|  | Q1 (*m/z*) | | Q3 (*m/z*) | | CE (eV) | | t_R_ (min) | |  |
| --- | --- | --- | --- | --- | --- | --- | --- | --- | --- |
| glyceric acid | | 240.0 | | 137.0 | | -20 | | 0.82 | |
| pyroglutamic acid | | 263.0 | | 137.0 | | -20 | | 1.90 | |
| lactic acid | | 224.0 | | 137.0 | | -20 | | 2.20 | |
| formic acid | | 180.1 | | 137.0 | | -20 | | 2.51 | |
| acetic acid | | 194.1 | | 137.0 | | -20 | | 2.62 | |
| propionic acid | | 208.1 | | 137.0 | | -20 | | 3.91 | |
| 4-hydroxyphenylacetic acid | | 286.1 | | 137.0 | | -25 | | 5.10 | |
| iso-butyric acid | | 222.1 | | 137.0 | | -20 | | 5.26 | |
| butyric acid | | 222.1 | | 137.0 | | -20 | | 5.42 | |
| 4-hydroxybenzoic acid | | 272.0 | | 137.0 | | -25 | | 5.44 | |
| 3-hydroxyphenylacetic acid | | 286.1 | | 137.0 | | -25 | | 5.61 | |
| 3-hydroxyglutaric acid (2 x D, singly charged) | | 417.0 | | 137.0 | | -25 | | 5.94 | |
| malic acid (2 x D, singly charged) | | 403.0 | | 137.0 | | -25 | | 6.02 | |
| 3-(3-hydroxyphenyl)propionic acid | | 300.1 | | 137.0 | | -25 | | 6.28 | |
| succinic acid (2 x D, singly charged) | | 387.0 | | 137.0 | | -25 | | 6.58 | |
| malonic acid (2 x D, singly charged) | | 373.0 | | 137.0 | | -25 | | 6.62 | |
| 2-methylbutyric acid | | 236.1 | | 137.0 | | -20 | | 6.69 | |
| iso-valeric acid | | 236.1 | | 137.0 | | -20 | | 6.87 | |
| valeric acid | | 236.1 | | 137.0 | | -20 | | 7.17 | |
| benzoic acid | | 256.0 | | 137.0 | | -20 | | 7.54 | |
| fumaric acid (2 x D, singly charged) | | 385.0 | | 137.0 | | -25 | | 7.65 | |
| phenylacetic acid | | 270.1 | | 137.0 | | -25 | | 7.91 | |
| 2-ethylbutyric acid | | 250.1 | | 137.0 | | -20 | | 7.94 | |
| 10-hydroxydecanoic acid | | 322.1 | | 137.0 | | -25 | | 8.19 | |
| 3,3-dimethylbutyric acid | | 236.1 | | 137.0 | | -20 | | 8.22 | |
| 2-methylvaleric acid | | 250.1 | | 137.0 | | -20 | | 8.35 | |
| 3-methylvaleric acid | | 250.1 | | 137.0 | | -20 | | 8.40 | |
| 4-methylvaleric acid | | 250.1 | | 137.0 | | -20 | | 8.62 | |
| hexanoic acid | | 250.1 | | 137.0 | | -20 | | 8.81 | |
| 3-phenylpropionic acid | | 284.1 | | 137.0 | | -25 | | 8.94 | |
| caprylic acid | | 278.1 | | 137.0 | | -25 | | 11.21 | |
| decanoic acid | | 306.1 | | 137.0 | | -25 | | 11.65 | |
| undecanoic acid | | 320.1 | | 137.0 | | -25 | | 11.79 | |
| lauric acid | | 334.1 | | 137.0 | | -25 | | 11.92 | |
| myristic acid | | 362.1 | | 137.0 | | -25 | | 12.23 | |
| linoleic acid | | 414.1 | | 137.0 | | -25 | | 12.35 | |
| pentadecanoic acid | | 376.1 | | 137.0 | | -25 | | 12.42 | |
| palmitic acid | | 390.1 | | 137.0 | | -25 | | 12.67 | |
| oleic acid | | 416.1 | | 137.0 | | -25 | | 12.70 | |
| stearic acid | | 418.1 | | 137.0 | | -25 | | 13.28 | |
| ^13^C2-acetic acid | | 196.1 | | 137.0 | | -20 | | 2.62 | |
| ^13^C3-propionic acid | | 211.1 | | 137.0 | | -20 | | 3.91 | |
| ^13^C4-butyric acid | | 226.1 | | 137.0 | | -20 | | 5.42 | |
| InjS-^13^C pyroglutamic acid | | 269.0 | | 143.0 | | -20 | | 1.90 | |
| InjS-^13^C formic acid | | 186.1 | | 143.0 | | -20 | | 2.51 | |
| InjS-^13^C acetic acid | | 200.1 | | 143.0 | | -20 | | 2.62 | |
| InjS-^13^C propionic acid | | 214.1 | | 143.0 | | -20 | | 3.91 | |
| InjS-^13^C 4-hydroxyphenylacetic acid | | 292.1 | | 143.0 | | -25 | | 5.10 | |
| InjS-^13^C iso-butyric acid | | 228.1 | | 143.0 | | -20 | | 5.26 | |
| InjS-^13^C butyric acid | | 228.1 | | 143.0 | | -20 | | 5.42 | |
| InjS-^13^C 3-hydroxyphenylacetic acid | | 292.1 | | 143.0 | | -25 | | 5.61 | |
| InjS-^13^C 3-(3-hydroxyphenyl)propionic acid | | 306.1 | | 143.0 | | -25 | | 6.28 | |
| InjS-^13^C 2-methylbutyric acid | | 242.1 | | 143.0 | | -20 | | 6.69 | |
| InjS-^13^C iso-valeric acid | | 242.1 | | 143.0 | | -20 | | 6.87 | |
| InjS-^13^C valeric acid | | 242.1 | | 143.0 | | -20 | | 7.17 | |
| InjS-^13^C phenylacetic acid | | 276.1 | | 143.0 | | -25 | | 7.91 | |
| InjS-^13^C 4-methylvaleric acid | | 256.1 | | 143.0 | | -20 | | 8.62 | |
| InjS-^13^C hexanoic acid | | 256.1 | | 143.0 | | -20 | | 8.81 | |
| InjS-^13^C 3-phenylpropionic acid | | 290.1 | | 143.0 | | -25 | | 8.94 | |
| InjS-^13^C myristic acid | | 368.1 | | 143.0 | | -25 | | 12.23 | |
| InjS-^13^C linoleic acid | | 420.1 | | 143.0 | | -25 | | 12.35 | |
| InjS-^13^C palmitic acid | | 396.1 | | 143.0 | | -25 | | 12.67 | |
| InjS-^13^C oleic acid | | 422.1 | | 143.0 | | -25 | | 12.70 | |

**Table S4**: Selected reaction monitoring (SRM) parameters for determination of aniline derivatives (positive ionization mode). The declustering potential was 70 eV for all compounds. CE: collision energy; t_R_: retention time; 2 x D: derivatized on 2 functional groups; n.d.: not detected; InjS-^13^C: injection standard prepared in-house with ^13^C-aniline; ^13^C_2_-acetic acid, ^13^C_3_-propionic acid and ^13^C_4_-butyric acid: fully labeled internal standards derivatized with native aniline.

|  | Q1 (*m/z*) | Q3 (*m/z*) | CE (eV) | t_R_ (min) |
| --- | --- | --- | --- | --- |
| glyceric acid | 182.0 | 94.0 | 30 | 0.85 |
| pyroglutamic acid | 205.0 | 94.0 | 30 | 1.77 |
| lactic acid | 166.0 | 94.0 | 30 | 2.54 |
| formic acid | 122.1 | 94.0 | 25 | 2.61 |
| acetic acid | 136.1 | 94.0 | 30 | 2.74 |
| propionic acid | 150.1 | 94.0 | 30 | 4.12 |
| 4-hydroxyphenylacetic acid | 228.1 | 94.0 | 30 | 5.63 |
| iso-butyric acid | 164.1 | 94.0 | 30 | 5.69 |
| butyric acid | 164.1 | 94.0 | 25 | 5.86 |
| 4-hydroxybenzoic acid | 214.0 | 94.0 | 30 | 5.88 |
| 3-hydroxyphenylacetic acid | 228.1 | 94.0 | 30 | 6.03 |
| 3-hydroxyglutaric acid (2 x D, singly charged) | 299.0 | 94.0 | 30 | 6.32 |
| malic acid (2 x D, singly charged) | 285.0 | 94.0 | 30 | 6.53 |
| 3-(3-hydroxyphenyl)propionic acid | 242.1 | 94.0 | 25 | 6.97 |
| 2-methylbutyric acid | 178.1 | 94.0 | 30 | 7.32 |
| succinic acid (2 x D, singly charged) | 269.0 | 94.0 | 30 | 7.47 |
| iso-valeric acid | 178.1 | 94.0 | 30 | 7.50 |
| malonic acid (2 x D, singly charged) | 255.0 | 94.0 | 30 | 7.86 |
| valeric acid | 178.1 | 94.0 | 25 | 7.95 |
| fumaric acid (2 x D, singly charged) | 267.0 | 94.0 | 30 | 8.61 |
| phenylacetic acid | 212.1 | 94.0 | 30 | 8.71 |
| 2-ethylbutyric acid | 192.1 | 94.0 | 25 | 8.72 |
| 3,3-dimethylbutyric acid | 178.2 | 94.0 | 30 | 9.07 |
| 10-hydroxydecanoic acid | 264.1 | 94.0 | 30 | 9.11 |
| 2-methylvaleric acid | 192.1 | 94.0 | 25 | 9.26 |
| 3-methylvaleric acid | 192.1 | 94.0 | 25 | 9.27 |
| 4-methylvaleric acid | 192.1 | 94.0 | 25 | 9.63 |
| hexanoic acid | 192.1 | 94.0 | 30 | 9.84 |
| 3-phenylpropionic acid | 226.1 | 94.0 | 35 | 9.87 |
| caprylic acid | 220.1 | 94.0 | 30 | 11.51 |
| decanoic acid | 248.1 | 94.0 | 30 | 11.86 |
| undecanoic acid | 262.1 | 94.0 | 30 | 12.01 |
| lauric acid | 276.1 | 94.0 | 30 | 12.18 |
| myristic acid | 304.1 | 94.0 | 35 | 12.60 |
| linoleic acid | 356.1 | 94.0 | 30 | 12.75 |
| pentadecanoic acid | 318.1 | 94.0 | 35 | 12.86 |
| palmitic acid | 332.1 | 94.0 | 35 | 13.19 |
| oleic acid | 358.1 | 94.0 | 35 | 13.25 |
| stearic acid | 360.1 | 94.0 | 35 | 14.09 |
| benzoic acid | 198.0 | 94.0 | 30 | n.d. |
| ^13^C2-acetic acid | 138.1 | 94.0 | 30 | 2.74 |
| ^13^C3-propionic acid | 153.1 | 94.0 | 30 | 4.12 |
| ^13^C4-butyric acid | 168.1 | 94.0 | 25 | 5.86 |
| InjS-^13^C pyroglutamic acid | 211.0 | 100.0 | 30 | 1.77 |
| InjS-^13^C formic acid | 128.1 | 100.0 | 25 | 2.61 |
| InjS-^13^C acetic acid | 142.1 | 100.0 | 30 | 2.74 |
| InjS-^13^C propionic acid | 156.1 | 100.0 | 30 | 4.12 |
| InjS-^13^C 4-hydroxyphenylacetic acid | 234.1 | 100.0 | 30 | 5.63 |
| InjS-^13^C iso-butyric acid | 170.1 | 100.0 | 30 | 5.69 |
| InjS-^13^C butyric acid | 170.1 | 100.0 | 25 | 5.86 |
| InjS-^13^C 3-hydroxyphenylacetic acid | 234.1 | 100.0 | 30 | 6.03 |
| InjS-^13^C 3-(3-hydroxyphenyl)propionic acid | 248.1 | 100.0 | 25 | 6.97 |
| InjS-^13^C 2-methylbutyric acid | 184.1 | 100.0 | 30 | 7.32 |
| InjS-^13^C iso-valeric acid | 184.1 | 100.0 | 30 | 7.50 |
| InjS-^13^C valeric acid | 184.1 | 100.0 | 25 | 7.95 |
| InjS-^13^C phenylacetic acid | 218.1 | 100.0 | 30 | 8.71 |
| InjS-^13^C 4-methylvaleric acid | 198.1 | 100.0 | 25 | 9.63 |
| InjS-^13^C hexanoic acid | 198.1 | 100.0 | 30 | 9.84 |
| InjS-^13^C 3-phenylpropionic acid | 232.1 | 100.0 | 35 | 9.87 |
| InjS-^13^C myristic acid | 310.1 | 100.0 | 35 | 12.60 |
| InjS-^13^C linoleic acid | 362.1 | 100.0 | 30 | 12.75 |
| InjS-^13^C palmitic acid | 338.1 | 100.0 | 35 | 13.19 |
| InjS-^13^C oleic acid | 364.1 | 100.0 | 35 | 13.25 |

**Table S5**: AIC-HR-MS parameters for determination of carboxylic acids. t_R_: retention time

|  | [M-H]^-^ | t_R_ (min) |
| --- | --- | --- |
| pyroglutamic acid | 128.0353 | 5.93 |
| glyceric acid | 105.0193 | 6.18 |
| lactic acid | 89.0244 | 6.25 |
| acetic acid | 59.0138 | 6.52 |
| propionic acid | 73.0295 | 6.98 |
| iso-butyric acid | 87.0451 | 7.42 |
| butyric acid | 87.0451 | 7.65 |
| 2-methylbutyric acid | 101.0608 | 8.16 |
| iso-valeric acid | 101.0608 | 8.36 |
| 2-ethylbutyric acid | 115.0764 | 8.88 |
| valeric acid | 101.0608 | 9.31 |
| 3,3-dimethylbutyric acid | 115.0764 | 9.41 |
| 2-methylvaleric acid | 115.0764 | 10.09 |
| 3-methylvaleric acid | 115.0764 | 10.33 |
| 4-methylvaleric acid | 115.0764 | 11.17 |
| hexanoic acid | 115.0764 | 12.20 |
| 3-hydroxyglutaric acid | 147.0299 | 15.55 |
| phenylacetic acid | 135.0451 | 15.66 |
| succinic acid | 117.0193 | 15.94 |
| malic acid | 133.0142 | 15.98 |
| malonic acid | 103.0037 | 16.45 |
| 10-hydroxydecanoic acid | 187.1339 | 17.72 |
| benzoic acid | 121.0295 | 17.74 |
| fumaric acid | 115.0037 | 18.09 |
| 3-phenylpropionic acid | 149.0608 | 18.85 |
| 4-hydroxyphenylacetic acid | 151.0400 | 20.73 |
| 4-hydroxybenzoic acid | 137.0244 | 21.02 |
| caprylic acid | 143.1077 | 21.60 |
| 3-hydroxyphenylacetic acid | 151.0400 | 21.68 |
| 3-(3-hydroxyphenyl)propionic acid | 165.0557 | 22.17 |
| ^13^C2-acetic acid | 61.0206 | 6.52 |
| ^13^C3-propionic acid | 76.0396 | 6.98 |
| ^13^C4-butyric acid | 91.0586 | 7.65 |

**Table S6**: Composition of the spiking solutions and spiked extracts for recovery determination in feces and ruminal fluid. Values are given for spiking level 1.

|  | Feces | | Ruminal fluid | |
| --- | --- | --- | --- | --- |
| Level 1 | c in spike solution (mg/L) | c spiked into extract (mg/L) | c in spike solution (mg/L) | c spiked into extract (mg/L) |
| acetic acid | 239 | 120 | 171 | 86 |
| propionic acid | 44 | 22 | 36 | 18 |
| butyric acid | 28 | 14 | 40 | 20 |
| linoleic acid | 19 | 9.4 |  |  |
| oleic acid | 9.7 | 4.9 |  |  |
| valeric acid | 6.3 | 3.1 | 4.2 | 2.1 |
| iso-butyric acid | 3.8 | 1.9 | 0.58 | 0.29 |
| 3-(3-hydroxyphenyl)propionic acid | 3.3 | 1.6 | 0.03 | 0.01 |
| myristic acid | 2.1 | 1.1 |  |  |
| 3-phenylpropionic acid | 2.0 | 1.0 | 3.1 | 1.5 |
| 2-methylbutyric acid | 1.6 | 0.82 | 1.2 | 0.60 |
| pyroglutamic acid | 1.3 | 0.66 | 0.30 | 0.15 |
| iso-valeric acid | 0.67 | 0.33 | 0.52 | 0.26 |
| 3-hydroxyphenylacetic acid | 0.34 | 0.17 | 0.04 | 0.02 |
| 4-hydroxyphenylacetic acid | 0.33 | 0.16 | 0.04 | 0.02 |
| 4-methylvaleric acid | 0.31 | 0.15 | 0.03 | 0.02 |
| palmitic acid | 0.29 | 0.15 |  |  |
| hexanoic acid | 0.17 | 0.09 | 2.1 | 1.0 |
| phenylacetic acid | 0.11 | 0.06 | 0.08 | 0.04 |

**Table S7**: Limits of detection (LOD) and limits of quantification (LOQ) of carboxylic acids present in feces and ruminal fluid. CO: could not be determined due to carry over; n.d.: not detected.

|  | Feces (mg/kg) | | | | | |
| --- | --- | --- | --- | --- | --- | --- |
|  | aniline derivatization | | 3-NPH derivatization | | AIC-HR-MS | |
|  | **LOD** | **LOQ** | **LOD** | **LOQ** | **LOD** | **LOQ** |
| acetic acid | 1.4 | 14 | 11 | 37 | 36 | 120 |
| propionic acid | 1.4 | 4.2 | 0.4 | 3.7 | 7.2 | 24 |
| butyric acid | 1.4 | 14 | 1.1 | 11 | 3.2 | 11 |
| valeric acid | 0.4 | 4.2 | 1.1 | 11 | 3.6 | 12 |
| hexanoic acid | 0.4 | 4.2 | 0.4 | 11 | 0.4 | 1.4 |
| pyroglutamic acid | 4.2 | 14 | 1.1 | 11 | CO | 3.6 |
| phenylacetic acid | 0.4 | 4.2 | 0.8 | 3.7 | 11 | 36 |
| 3-phenylpropionic acid | 0.4 | 4.2 | 0.4 | 1.3 | 1.8 | 6.1 |
| 4-hydroxyphenylacetic acid | 0.4 | 1.4 | 1.1 | 3.7 | 4.3 | 22 |
| 3-(3-hydroxyphenyl)propionic acid | 0.5 | 1.4 | 1.1 | 3.7 | 6.5 | 22 |
| iso-butyric acid | 2.0 | 5.9 | 11 | 37 | 4.3 | 14 |
| 2-methylbutyric acid | 1.4 | 4.2 | 1.1 | 3.7 | 3.6 | 12 |
| 4-methylvaleric acid | 0.4 | 1.4 | 1.1 | 3.7 | 1.8 | 5.8 |
| 3-hydroxyphenylacetic acid | 1.4 | 4.2 | 2.5 | 8.8 | 18 | 58 |
| iso-valeric acid | 0.4 | 4.2 | 1.1 | 11 | 3.6 | 12 |
| myristic acid | CO | 4.2 | 1.1 | 3.7 | n.d. | n.d. |
| palmitic acid | CO | 42 | 3.7 | 37 | n.d. | n.d. |
| linoleic acid | CO | 14 | 11 | 37 | n.d. | n.d. |
| oleic acid | CO | 14 | 11 | 37 | n.d. | n.d. |
|  | Ruminal fluid (mg/L) | | | | | |
|  | aniline derivatization | | 3-NPH derivatization | | AIC-HR-MS | |
|  | **LOD** | **LOQ** | **LOD** | **LOQ** | **LOD** | **LOQ** |
| acetic acid | 2.0 | 20 | 15 | 51 | 50 | 167 |
| propionic acid | 2.0 | 5.9 | 0.5 | 5.1 | 10 | 34 |
| butyric acid | 2.0 | 20 | 1.6 | 15 | 4.5 | 15 |
| valeric acid | 0.6 | 5.9 | 1.6 | 15 | 5.0 | 17 |
| hexanoic acid | 0.6 | 5.9 | 0.5 | 15 | 0.5 | 2.0 |
| pyroglutamic acid | 5.9 | 20 | 1.6 | 15 | CO | 5.0 |
| phenylacetic acid | 0.6 | 5.9 | 1.1 | 5.1 | 15 | 50 |
| 3-phenylpropionic acid | 0.6 | 5.9 | 0.5 | 1.8 | 2.5 | 8.5 |
| 4-hydroxyphenylacetic acid | 0.6 | 2.0 | 1.6 | 5.1 | 6.0 | 30 |
| 3-(3-hydroxyphenyl)propionic acid | 0.7 | 2.0 | 1.6 | 5.1 | 9.0 | 30 |
| iso-butyric acid | 2.7 | 8.2 | 15 | 51 | 6.0 | 20 |
| 2-methylbutyric acid | 2.0 | 5.9 | 1.6 | 5.1 | 5.0 | 17 |
| 4-methylvaleric acid | 0.6 | 2.0 | 1.6 | 5.1 | 2.5 | 8.0 |
| 3-hydroxyphenylacetic acid | 2.0 | 5.9 | 3.5 | 12 | 25 | 80 |
| iso-valeric acid | 0.6 | 5.9 | 1.6 | 15 | 5.0 | 17 |
| myristic acid | CO | 5.9 | 1.6 | 5.1 | n.d. | n.d. |
| palmitic acid | CO | 59 | 5.1 | 51 | n.d. | n.d. |
| linoleic acid | CO | 20 | 15 | 51 | n.d. | n.d. |
| oleic acid | CO | 20 | 15 | 51 | n.d. | n.d. |

**Table S8**: Relative standard deviations (n=4, values in %) of sample work-up and measurement for carboxylic acids detected in feces and ruminal fluid as well as for the added ^13^C-labelled internal standards. Aniline: compounds derivatized with aniline and measured by LC-MS/MS; 3-NPH: compounds derivatized with 3-NPH and measured by LC-MS/MS; < LOQ: below limit of quantification, CO: not determined because of carry over (same peak areas as in derivatized blanks), n.d.: not detected.

|  | Feces | | | Ruminal fluid | | |
| --- | --- | --- | --- | --- | --- | --- |
|  | aniline | 3-NPH | AIC-HR-MS | aniline | 3-NPH | AIC-HR-MS |
| acetic acid | 5.5 | 5.1 | 5.6 | 6.1 | 11 | 2.5 |
| propionic acid | 5.3 | 8.3 | 6.6 | 7.4 | 4.7 | 3.4 |
| butyric acid | 6.1 | 8.6 | 5.4 | 9.0 | 5.0 | 2.8 |
| valeric acid | 4.7 | 8.8 | 6.6 | 11 | 3.4 | 3.5 |
| hexanoic acid | 6.7 | 41 | 16 | 7.2 | 4.9 | 2.4 |
| pyroglutamic acid | 16 | 4.5 | 5.3 | < LOQ | 14 | 5.4 |
| phenylacetic acid | 13 | 10 | < LOQ | 13 | 15 | < LOQ |
| 3-phenylpropionic acid | 10 | 7.1 | 7.7 | 7.9 | 3.9 | 2.1 |
| 4-hydroxyphenylacetic acid | 18 | 12 | 4.4 | n.d. | n.d. | n.d. |
| 3-(3-hydroxyphenyl)propionic acid | 7.6 | 5.6 | 12 | n.d. | n.d. | n.d. |
| iso-butyric acid | 7.0 | 31 | 10 | 22 | 6.1 | 6.8 |
| 2-methylbutyric acid | 5.8 | 10 | 13 | 23 | 3.7 | 12 |
| 4-methylvaleric acid | 6.1 | 8.5 | 12 | n.d. | n.d. | n.d. |
| 3-hydroxyphenylacetic acid | 26 | 20 | n.d. | n.d. | n.d. | n.d. |
| iso-valeric acid | 6.3 | 5.1 | 18 | 15 | 9.3 | 4.7 |
| myristic acid | 7.2 | 9.1 | n.d. | n.d. | n.d. | n.d. |
| palmitic acid | 19 | n.d. | n.d. | n.d. | n.d. | n.d. |
| linoleic acid | 12 | 8.6 | n.d. | n.d. | n.d. | n.d. |
| oleic acid | 11 | 18 | n.d. | n.d. | n.d. | n.d. |
| ^13^C-acetic acid | 2.6 | 9.1 | 2.0 | 5.5 | 4.4 | 1.6 |
| ^13^C-propionic acid | 3.3 | 3.1 | 2.0 | 6.3 | 3.3 | 1.4 |
| ^13^C-butyric acid | 3.6 | 0.8 | 2.1 | 10 | 2.8 | 2.0 |

**Table S9**: Relative standard deviations (n=2, values in %) of sample work-up and measurement of feces used for validation determined by external calibration curve (Ex cal) and by standard addition method (SA). Phenylacetic acid, 4-methylvaleric acid and 3-hydroxyphenylacetic acid occurred in traces, whereas palmitic acid was below the LOD in all methods. n.a.: not assessed. This table directly relates to Table 4 in the main manuscript.

|  | aniline derivatization | | 3-NPH derivatization | | AIC-HR-MS | |
| --- | --- | --- | --- | --- | --- | --- |
|  | Ex cal | SA | Ex cal | SA | Ex cal | SA |
| acetic acid | 1.8 | 3.9 | 4.8 | 4.4 | 3.7 | 0.6 |
| propionic acid | 5.2 | 5.9 | 0.4 | 0.6 | 3.4 | 3.6 |
| butyric acid | 5.8 | 1.0 | 0.8 | 3.9 | 5.2 | 0.9 |
| valeric acid | 3.3 | 7.3 | 6.3 | 6.2 | 2.5 | 2.9 |
| hexanoic acid | n.a. | n.a. | n.a. | n.a. | 18 | 1.2 |
| pyroglutamic acid | 10 | 44 | 3.4 | 8.7 | 3.4 | 3.7 |
| 3-phenylpropionic acid | 2.8 | 0.5 | 3.6 | 5.3 | 2.3 | 1.7 |
| 4-hydroxyphenylacetic acid | n.a. | n.a. | 28 | 18 | n.a. | n.a. |
| 3-(3-hydroxy-phenyl)propionic acid | 6.4 | 14 | 0.4 | 3.0 | 13 | 6.0 |
| iso-butyric acid | 6.1 | 36 | 8.7 | 2.2 | 6.4 | 0.5 |
| 2-methylbutyric acid | n.a. | n.a. | 1.3 | 11 | 14 | 23 |
| iso-valeric acid | n.a. | n.a. | 17 | 10 | 57 | 14 |
| myristic acid | 0.9 | 41 | 0.1 | 26 | n.a. | n.a. |
| linoleic acid | 1.7 | 4.5 | 11 | 0.5 | n.a. | n.a. |
| oleic acid | 18 | 18 | 5.6 | 37 | n.a. | n.a. |

**Table S10**: Relative standard deviations (n=2, values in %) of sample work-up and measurement of ruminal fluid used for validation determined by external calibration curve (Ex cal) and by standard addition method (SA). Phenylacetic acid, 4-methylvaleric acid and 3-hydroxyphenylacetic acid occurred in traces, whereas 4-hydroxyphenylacetic acid, 3-(3-hydroxy-phenyl)propionic acid, 4-methylvaleric acid, 3-hydroxyphenylacetic acid and all investigated long chain fatty acids were below the LOD in all methods. n.a.: not assessed. This table directly relates to Table 5 in the main manuscript.

|  | aniline derivatization | | 3-NPH derivatization | | AIC-HR-MS | |
| --- | --- | --- | --- | --- | --- | --- |
|  | Ex cal | SA | Ex cal | SA | Ex cal | SA |
| acetic acid | 4.6 | 10 | 3.4 | 3.3 | 1.6 | 6.4 |
| propionic acid | 7.1 | 9.6 | 1.0 | 3.2 | 0.1 | 3.3 |
| butyric acid | 6.9 | 15 | 1.7 | 0.9 | 1.4 | 3.3 |
| valeric acid | 4.8 | 6.9 | 1.9 | 8.8 | 1.6 | 5.6 |
| hexanoic acid | 5.5 | 7.2 | 14 | 4.2 | 5.9 | 2.8 |
| pyroglutamic acid | n.a. | n.a. | n.a. | n.a. | n.a. | 3.3 |
| 3-phenylpropionic acid | 15 | 10 | 3.3 | 9.8 | 2.3 | 2.4 |
| iso-butyric acid | 4.3 | 16 | 7.2 | 8.1 | 5.9 | 52 |
| 2-methylbutyric acid | n.a. | n.a. | 0.2 | 5.6 | 22 | 14 |
| iso-valeric acid | 0.1 | 8.8 | 14 | 12 | 2.5 | 22 |

References:

1. Chan JC, Kioh DY, Yap GC, Lee BW, Chan EC. A novel LCMSMS method for quantitative measurement of short-chain fatty acids in human stool derivatized with (12)C- and (13)C-labelled aniline. J Pharm Biomed Anal. 2017;138:43-53. <https://doi.org/10.1016/j.jpba.2017.01.044>.
